# Combined inhibition of BETs and HDACs as a potential epigenetics-based therapy for malignant rhabdoid tumor

**DOI:** 10.1038/s41419-026-09026-z

**Published:** 2026-06-22

**Authors:** Lingyan Zhu, Yuanyuan Li, Yaxian Wen, Jinxuan Wen, Junzhu Zhang, Lingzhao Min, Changsheng Li, Qi Peng, Lisha Wang, Anqi Li, Zebing Liu, Xiaoqiang Wang

**Affiliations:** 1 Department of Pathology, Renji Hospital, School of Medicine, Shanghai Jiaotong University, Shanghai, China; 2 Department of Pediatric Neurosurgery, Xinhua Hospital Affiliated to Shanghai Jiaotong University School of Medicine, Shanghai, China; 3 Department of Pathology, Beth Israel Deaconess Medical Center, Harvard Medical School, Boston, MA USA; 4 Department of Pathology, Ruijin Hospital, School of Medicine, Shanghai Jiaotong University, Shanghai, China

**Keywords:** Paediatric cancer, Sarcoma

## Abstract

Malignant rhabdoid tumors (MRT) are highly aggressive pediatric malignancies driven by *SMARCB1* loss and consequent epigenetic dysregulation, with dismal survival rates underscoring the need for novel therapies. Given the central role of chromatin modifiers in MRT pathogenesis, we investigated a novel combinatorial epigenetic approach simultaneously targeting histone deacetylases (HDACs) and bromodomain and extraterminal (BET) family proteins. Using a focused epigenetic compound screen across multiple MRT cell lines, we identified that the HDAC inhibitor panobinostat (LBH589) and the BET inhibitor birabresib (OTX015) act synergistically to inhibit cell proliferation, with combination index (CI) values consistently <1. Transcriptomic profiling revealed that this synergy is underpinned by a profound downregulation of cell cycle genes, leading to G1/S phase arrest. Mechanistically, the combination therapy cooperatively restores the expression of the cyclin-dependent kinase inhibitors p21 and p16. This restoration inhibits cyclin D1–CDK4/6 kinase activity, reduces retinoblastoma protein (Rb) phosphorylation, and consequently represses the E2F1 transcriptional program and its key targets, including cyclin A2 and cyclin B1. In vivo, this dual-epigenetic targeting significantly attenuated tumor growth in MRT xenograft models, outperforming either monotherapy, and was associated with suppressed proliferation and E2F1 signaling. Our findings unveil a novel synergistic strategy that pharmacologically recapitulates a core SMARCB1-mediated tumor-suppressive circuit, nominating combined HDAC and BET inhibition as a promising therapeutic avenue for MRT.

## Introduction

Malignant rhabdoid tumor (MRT) is a rare and highly aggressive malignancy with poor prognoses [1, 2]. Predominantly affecting infants and young children, MRT arises primarily in the kidneys and central nervous system. Current multimodal regimens involve early surgical resection, intensive multi-agent chemotherapy, radiotherapy to involved sites, and/or high-dose chemotherapy with autologous stem-cell rescue. Despite these interventions, the 5-year survival rate remains below 30% [3], underscoring an urgent need for novel therapeutic strategies.

MRT is genetically defined by biallelic inactivation of *SMARCB1* (or rarely, *SMARCA4*), encoding SNF5 (also known as INI1 or BAF47), a core subunit of SWI/SNF chromatin remodeling complex. Beyond this hallmark alteration, MRT genomes exhibit remarkable stability, harboring negligible recurrent mutations and displaying the lowest mutational burden among sequenced cancer types [4]. Murine models confirm that *Smarcb1* inactivation drives tumorigenesis [5], establishing SMARCB1 as a bona fide tumor suppressor and implicating SWI/SNF-dependent epigenetic dysregulation as central to MRT pathogenesis.

Epigenetic regulation—mediated by DNA methylation, non-coding RNAs, and histone modifications—orchestrates chromatin accessibility and gene expression. Histone acetylation, dynamically controlled by histone acetyltransferases (HATs) and histone deacetylases (HDACs), modulates chromatin architecture and transcriptional programs critical for cell cycle progression and differentiation. HATs (“writers”) deposit acetyl groups to relax chromatin and activate transcription, while HDACs (“erasers”) remove these marks to enforce repression. Aberrant HDAC activity in cancer frequently silences tumor suppressors or activates oncogenes [6]. In MRT, overexpression of multiple HDACs [7, 8] renders histone deacetylation a compelling therapeutic target. Preclinical studies demonstrate that HDAC inhibitors (HDACi) restore acetylation, mimicking SWI/SNF function in *SMARCB1*-deficient cells [9], inducing cell cycle arrest, apoptotic cell death, and terminal differentiation [7, 8, 10–12].

HDAC inhibition broadly elevates genomic acetylation, including at intergenic and gene body regions [13]. This hyperacetylation recruits Bromodomain and Extraterminal (BET) family proteins, consist of BRDT, BRD2, BRD3, and BRD4, which act as “chromatin readers” that localize to acetylated promoters and super-enhancers. BET proteins recruit transcriptional machinery to drive oncogene expression [14]. Targeting BET proteins represents a promising therapeutic strategy in oncology due to their central role in oncogenic transcription. Mechanistically, HDAC and BET inhibitors exhibit complementary actions: HDAC inhibition increases histone acetylation, thereby enhancing BET protein recruitment to chromatin, while BET inhibitors block downstream transcriptional activation. Synergistic antitumor effects of combined HDAC-BET inhibition have been empirically validated across diverse malignancies—including pancreatic cancer [15], breast cancer [16], rhabdomyosarcoma [17], and lymphoma [18]. Given this compelling preclinical evidence and the critical dependence of MRT on epigenetic dysregulation, it is worthy to explore whether dual HDAC/BET targeting would demonstrate synergistic anti-proliferative efficacy against MRT.

SMARCB1-mediated transcriptional regulation of cell cycle genes is well-established through cellular and in vivo models. Reconstitution of functional SMARCB1 induces G1 cell cycle arrest concomitant with upregulation of p21 and p16 (encoded by *CDKN1A* and *CDKN2A*, respectively) [19–22]. Furthermore, SMARCB1 activation may enforce the mitotic checkpoint via the p16-Cyclin D1/CDK4-pRb-E2F axis [23]. Here, we demonstrate that pharmacologic co-inhibition of HDAC and BET proteins by panobinostat (LBH589) and birabresib (OTX015) synergistically suppresses rhabdoid tumor cell proliferation by recapitulating this regulatory circuit. Mechanistic analysis revealed that combination treatment induces G1 phase arrest through coordinated activation of p16/p21-cyclin D1-CDK4/6-pRb-E2F signaling—phenocopying key aspects of SMARCB1-mediated cell cycle control. This potent synergy highlights the therapeutic potential of epigenetic dual-targeting strategies for rhabdoid tumors.

## Results

### Synergistic effects of the HDAC inhibitor LBH589 and the BET inhibitor OTX015

**Fig. 1:**
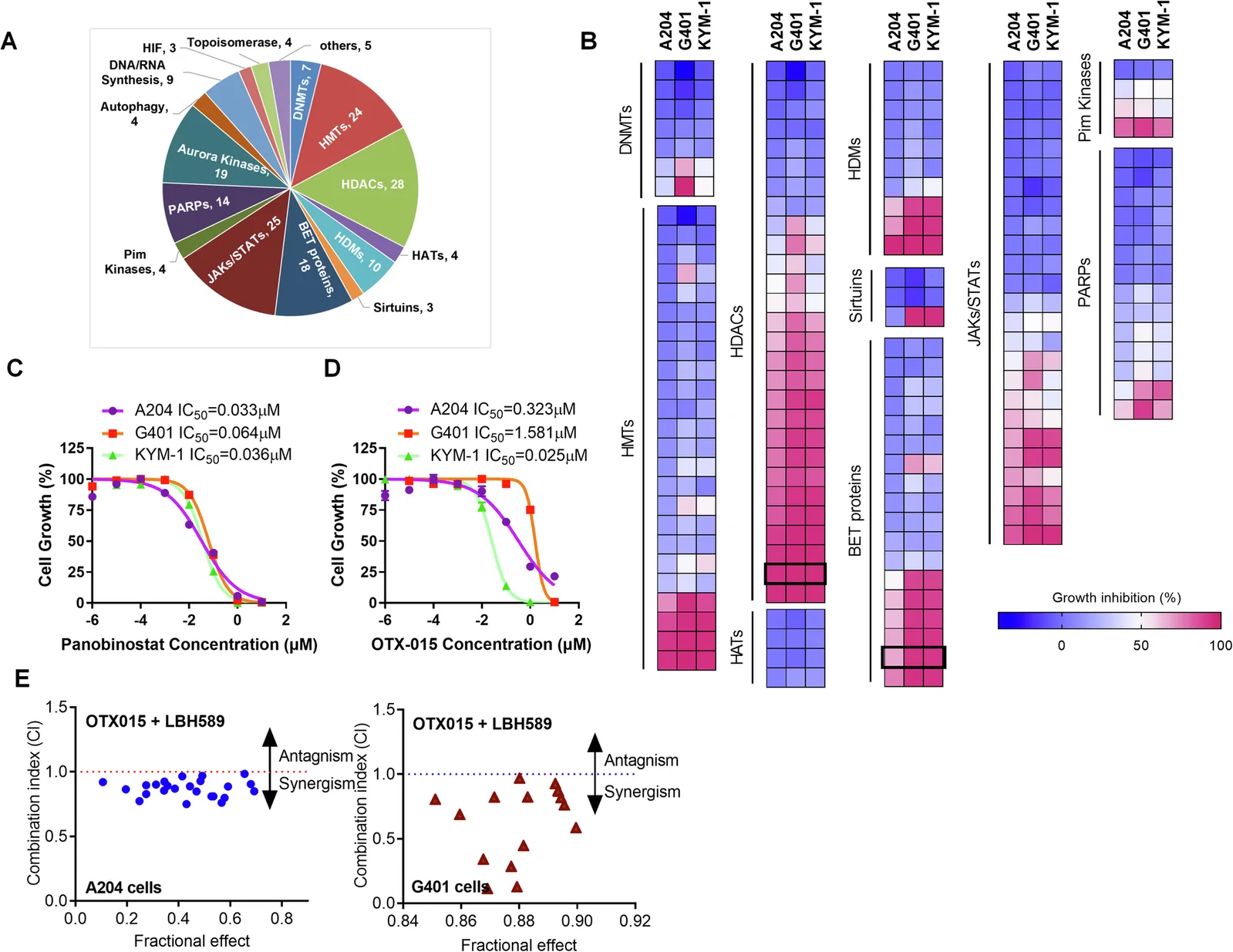
Combined HDAC and BET inhibition synergistically suppresses MRT cell growth. **A** Pie chart showing the classification by compound type. **B** Heatmap depicting results from an epigenetic compound library screen (181 compounds at 2.5 µM for 72 h) in three MRT cell lines. Blue and purple represent cell growth inhibition and induction, respectively. Dose-response curves for LBH589 (**C**) and OTX015 (**D**) monotherapy. Cell viability was measured after 72 h (mean ± SD, *n* = 3). **E** Synergy analysis of LBH589 and OTX015 combination. Combination index (CI) values were calculated using the Chou–Talalay method from viability data (AlamarBlue assay, 72 h treatment) in A204 and G401 cells. CI < 1 indicates synergy. Data are representative of three independent experiments.

An epigenetic compound screen across three MRT cell lines (A204, G401, KYM-1) identified multiple drug classes—including HDAC and BET inhibitors—that suppressed proliferation in all models (Fig. 1A, B). Subsequent dose-response analysis revealed concentration-dependent growth inhibition by LBH589 (a potent HDAC inhibitor) and OTX015 (a high-affinity BET inhibitor) in MRT cells, as quantified via alamarBlue assays (Fig. 1C, D). Synergy assessment using the Chou-Talalay median-effect principle demonstrated significantly enhanced efficacy with combination treatment. Co-administration of LBH589 and OTX015 generated combination index (CI) values substantially below 1.0 in both A204 and G401 cell lines (*P* < 0.01 versus monotherapies; Fig. 1E), confirming synergistic cytotoxicity against MRT cells.

### LBH589 and OTX015 mainly affects cell cycle in MRT

**Fig. 2:**
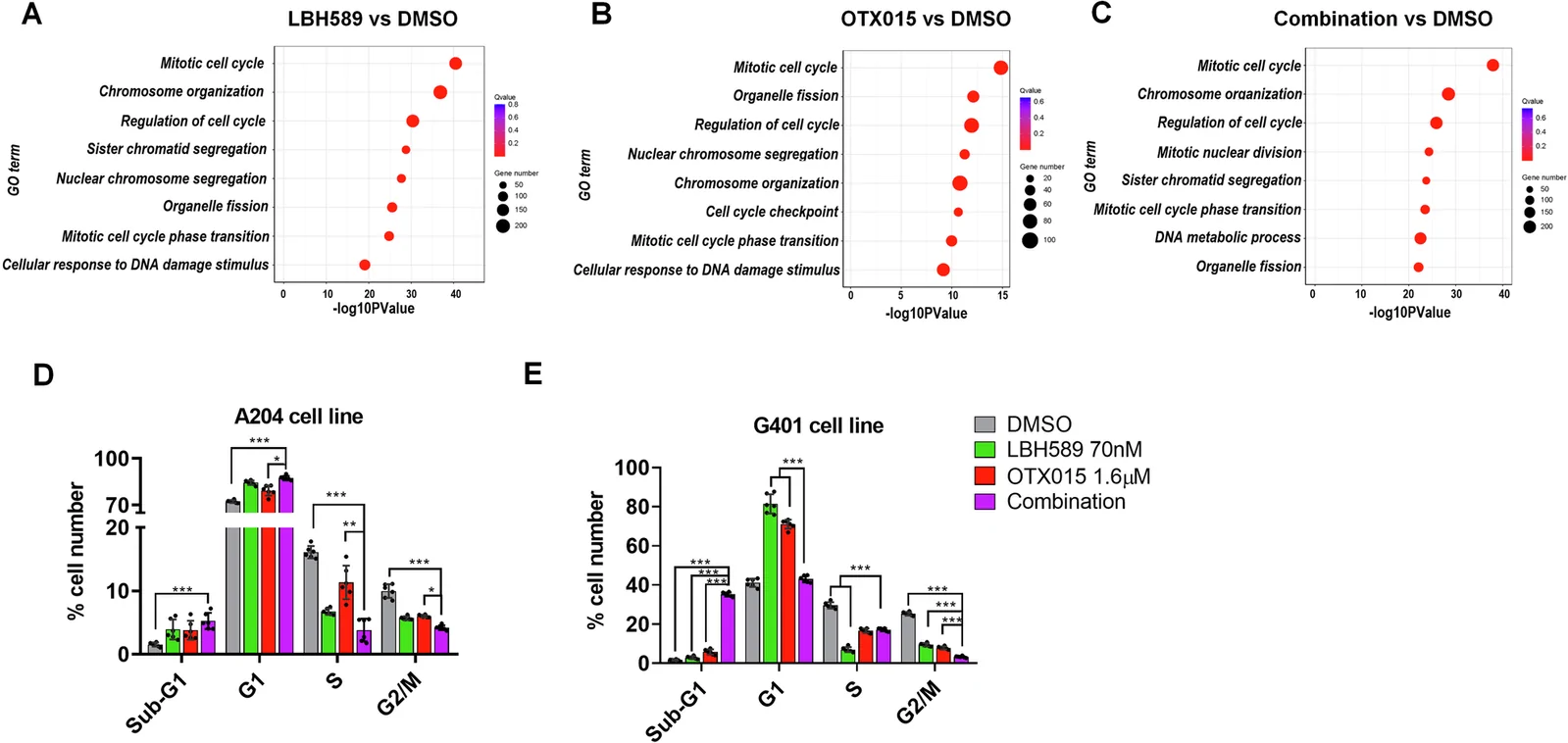
Combined BET and HDAC inhibition induces G₁-phase arrest in MRT cells. **A**–**C** Gene set enrichment analysis (GSEA) of RNA-sequencing data from A204 cells treated for 16 h with DMSO (control), LBH589, OTX015, or their combination. Enrichment plots show pathways significantly downregulated by LBH589 (**A**), OTX015 (**B**), or the combination (**C**). NES, normalized enrichment score. Cell cycle distribution analyzed by flow cytometry in A204 (**D**) and G401 (**E**) cells after 48 h treatment. Bar graphs show the percentage of cells in each phase (mean ± SD, *n* = 6). Statistical significance was determined using the Kruskal–Wallis test followed by Dunn’s multiple comparisons test. *P < 0.05, **P < 0.01, ***P < 0.001 vs. DMSO control (two-tailed Student’s *t*-test). Data are representative of three independent experiments.

To elucidate the mechanistic basis of LBH589/OTX015 synergy, we performed RNA-sequencing (RNA-seq) on A204 cells treated with: (i) panobinostat (LBH589), (ii) birabresib (OTX015), or (iii) combination therapy. Principal component analysis demonstrated clear segregation of treatment groups from DMSO controls (Fig. S1A), indicating genome-wide transcriptional reprogramming. Notably, 48.5% of differentially expressed genes in OTX015-treated cells overlapped with LBH589-altered transcripts (Fig. S1B, C), implying convergent epigenetic regulation. Gene set enrichment analysis (GSEA) further identified significant suppression of mitotic cell cycle pathways—including chromosome segregation, organelle fission, and nuclear division—across all treatment arms (Fig. 2A–C). Consistently, key S/G2-M phase regulators showed coordinated downregulation (Fig. S1D, E), with parallel observations in G401 cells (Fig. S2A–E).

Cell cycle profiling by flow cytometry confirmed these findings. In A204 cells, LBH589 monotherapy and combination treatment induced comparable in G1/S phase arrest, which was more pronounced than with OTX015 alone (Fig. 2D). While in G401 cells, all drug-treated groups exhibited markedly enhanced G1 phase arrest relative to the DMSO-treated controls. Furthermore, the combination of LBH589 and OTX015 resulted in a substantial increase in the sub-G1 population, indicative of apoptotic cells (Fig. 2E). While neither cleaved caspase-3 nor cleaved PARP1 was detectable under any treatment condition in A204 cells, the combination treatment induced a strong cleaved PARP1 signal and a weak but reproducible cleaved caspase-3 band (Fig. S3), suggesting that apoptosis contributes to the growth inhibition in G401, albeit to a lesser extent than cell cycle arrest. These data establish cell cycle blockade as the primary mechanism underlying MRT growth suppression by dual HDAC/BET inhibition.

### BET and HDAC inhibitors synergistically suppress E2F1

**Fig. 3:**
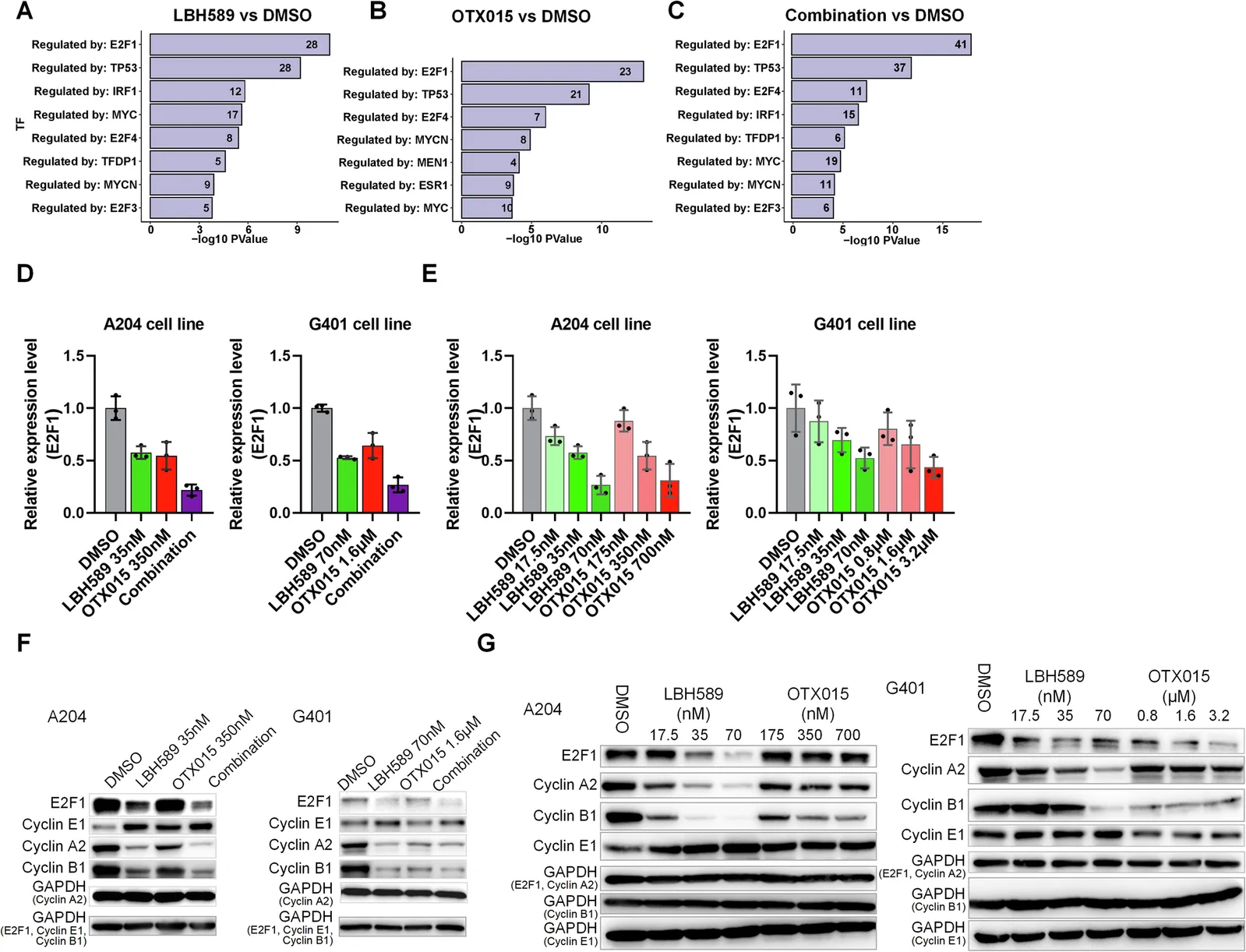
Dual HDAC and BET inhibition cooperatively suppresses E2F1. Transcription factor motif enrichment analysis (GSEA) in A204 cells treated for 16 h with LBH589 (**A**), OTX015 (**B**), or their combination (**C**), compared to DMSO control. **D**, **E** *E2F1* mRNA levels measured by RT-qPCR in A204 (left) and G401 (right) cells after 16 h treatment. **D** Cells treated with DMSO, LBH589, OTX015, or the combination. **E** Cells were treated with DMSO, indicated dose of LBH589 or OTX015. **F** A204 and G401 cells were treated with the indicated concentrations of LBH589 or OTX015, alone or in combination, for 24 h. Whole-cell lysates were then prepared and subjected to Western blot analysis to assess the protein expression levels of E2F1, cyclin E1, cyclin A2, cyclin B1, and GAPDH. **G** Dose-dependent downregulation of E2F1 and cyclin proteins. A204 cells were treated with LBH589 (17.5, 35, and 70 nM) and OTX015 (175, 350, and 700 nM). G401 cells with LBH589 (17.5, 35, and 70 nM) and OTX015 (0.8, 1.6, and 3.2 μM). Blots are representative of two independent experiments.

To delineate the molecular mechanisms underlying the synergistic antitumor effects of LBH589/OTX015 in MRT cells, we performed transcription factor motif enrichment analysis via GSEA. This revealed significant enrichment of E2F1, TP53, MYC, E2F4, and MYCN binding motifs in promoters modulated by LBH589, OTX015, or their combination in A204 cells (Fig. 3A–C). Similar enrichment patterns for E2F1, TP53, E2F4, and MYCN were observed in G401 cells (Fig. S2F, G).

Correlated with our observed G1 arrest (Fig. 2D, E), we identified the functionally well-characterized E2F1 as the most significantly enriched transcription factor (Fig. 3A–C), which serves as a master regulator of critical cellular processes, including cell cycle progression, DNA damage response, and apoptosis [24]. Notably, Combination therapy substantially reduced expression of *E2F1* and its core cell cycle targets, such as *CCNA2* (encoding cyclin A2), *CCNB1* (encoding cyclin B1), at both transcript and protein levels (Figs. 3D–G, S4 and S5), exhibiting dose-dependent suppression of these effectors (Fig. 3E, G).

Following the treatment with LBH589, the chromatin accessibility underwent a significant change, while OTX015 had a negligible effect, as expected. Moreover, the combination treatment provided transcriptional access to significantly more genes than LBH589 treatment alone (Fig. S6A, B). Additionally, the genomic regions of *E2F1*, *CCNA2*, and *CCNB1* exhibited decreased chromatin accessibility when treated with LBH589 alone or in combination with OTX015 (Fig. S6C). Importantly, the levels of newly synthesized nascent RNA of *E2F1*, *CCNA2*, and *CCNB1* were decreased with either LBH589 or OTX015 treatment compared with the DMSO treatment control, and the combined treatment had a synergistic effect on the transcription of these genes (Fig. S6D, E). These data suggest that LBH589/OTX015 transcriptionally suppressed *E2F1*.

### Co-targeting HDAC and BET induces cooperative repression of the CDK4/6-Rb-E2F axis

**Fig. 4:**
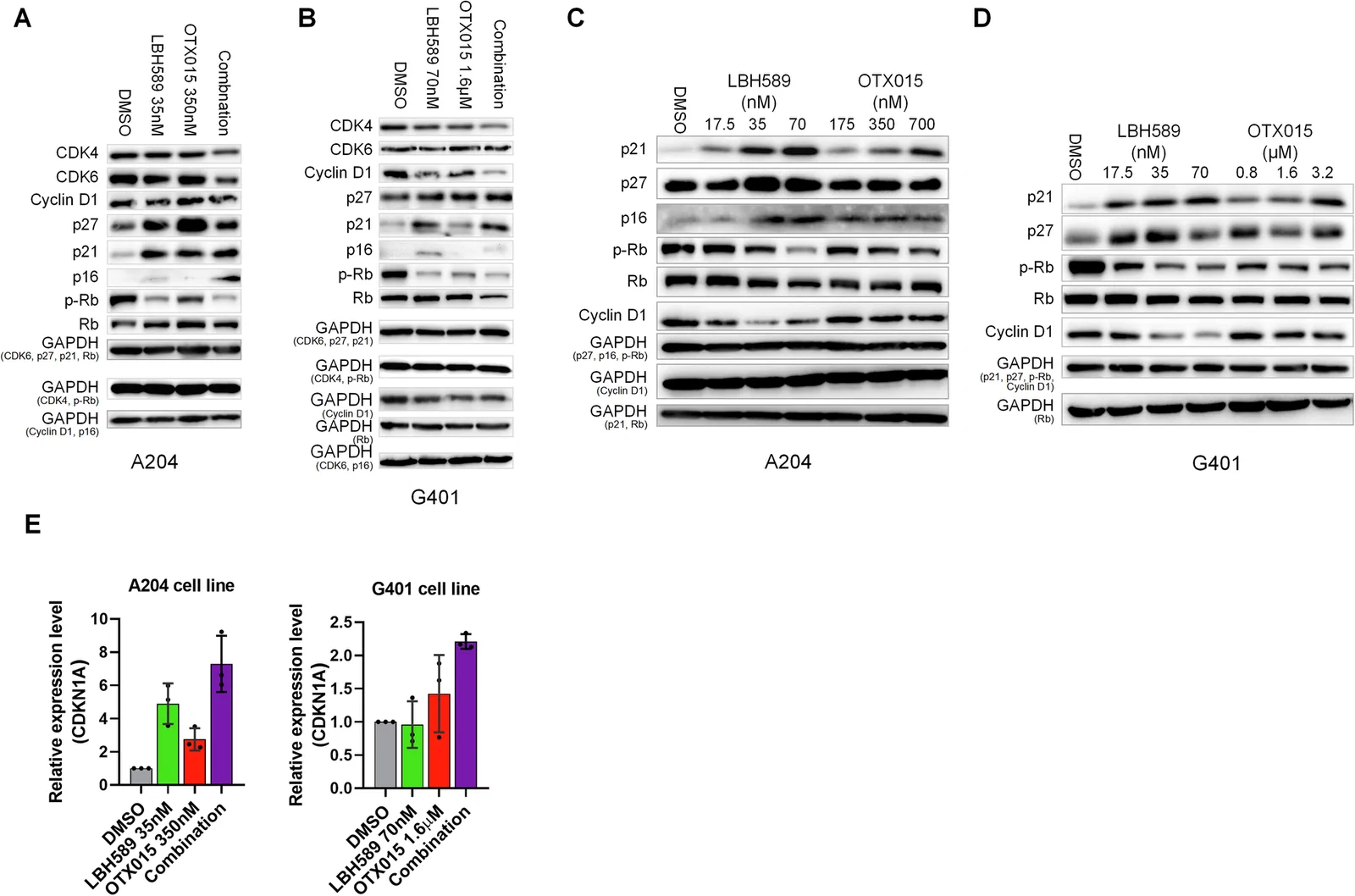
Synergistic effects of BET and HDAC dual-inhibition in blocking CDK4/6-Rb-E2F axis. Immunoblot analysis of key regulators in the CDK4/6–Rb pathway in A204 (**A**) and G401 (**B**) cells treated for 24 h with indicated concentrations of LBH589, OTX015, or their combination. Protein levels of CDK4, CDK6, cyclin D1, p21, p27, p16, total Rb, phosphorylated Rb (p-Rb), and GAPDH (loading control) are shown. **C**, **D** Dose-dependent modulation of the pathway. **C** A204 treated with LBH589 (17.5, 35, and 70 nM) and OTX015 (175, 350, and 700 nM). **D** G401 treated with LBH589 (17.5, 35, and 70 nM) and OTX015 (0.8, 1.6, and 3.2 μM). Blots are representative of at least two independent experiments. **E** Quantification of nascent *CDKN1A* mRNA in LBH589- and/or OTX015-treated A204 and G401 cells.

E2F1 activity is physiologically restrained by pocket proteins, with Rb being the most extensively characterized member that exhibits preferential binding affinity for E2F1. Sequential phosphorylation of Rb by cyclin D–CDK4/6 and cyclin E–CDK2 complexes at multiple serine and threonine residues triggers the release of E2F1 from the Rb pocket, thereby enabling its transcriptional activation [24]. Consistent with this mechanism, combined LBH589/OTX015 treatment significantly reduced the expression of CDK4/6 and cyclin D1, along with phospho-Rb levels, compared to single-agent treatment in both A204 and G401 cells (Fig. 4A, B). Moreover, treatment with LBH589 or OTX015 markedly upregulated p16, p21, and p27 (encoded by *CDKN1B*), which act as endogenous inhibitors of cyclin D1–CDK4/6 and cyclin E–CDK2 complex formation, thereby preventing Rb phosphorylation (Fig. 4A–D). The increased level of nascent RNA of *CDKN1A* in the combined treatment group indicates a synergistic effect of LBH589 and OTX015 in the transcriptional restoration of *CDKN1A* (Fig. 4E). Collectively, these results demonstrate that LBH589 and OTX015 synergistically inhibit cell cycle progression through suppression of the CDK4/6–Rb–E2F pathway.

### LBH589/OTX015 synergistically inhibits the growth of MRT cell line xenografts in vivo

**Fig. 5:**
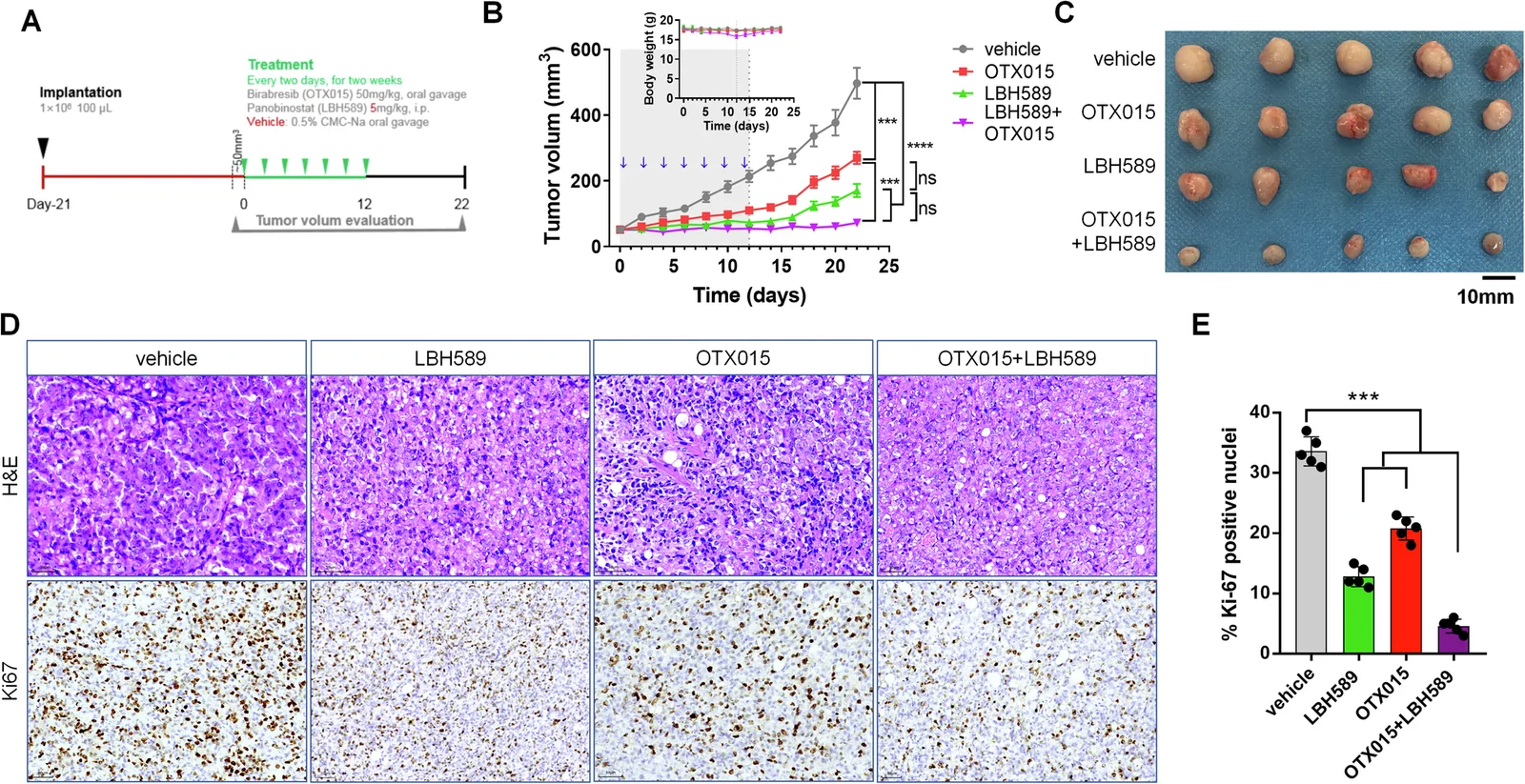
Dual HDAC and BET inhibition synergistically suppresses MRT growth in vivo. **A** Schematic of the in vivo treatment regimen. **B** Tumor growth curves of A204 xenografts in each treatment group (*n* = 5). Arrows indicate time points of drug administration. Data points represent mean tumor volume ± SD. The inset shows corresponding body weight changes (mean ± SD). **C** Representative images of excised tumors from each group at the study endpoint. **D** Representative H&E staining and immunohistochemistry for the proliferation marker Ki67 in tumor sections. Scale bars, 50 µm. **E** Quantification of Ki67-positive cells. Combined treatment significantly reduced tumor cell proliferation. Data are mean ± SD (*n* = 5). Statistical significance was determined by one-way ANOVA with Tukey’s multiple comparisons test; ns, not significant; ***P < 0.001; ****P < 0.0001.

To evaluate the therapeutic potential in vivo, A204 cells derived xenografts were established in NSG mice. Tumor-bearing mice were randomized into four groups as described in the Methods. By day 12, tumor growth was significantly suppressed in all treatment groups (LBH589 or OTX015 monotherapy, and their combination) compared to the vehicle control (Fig. 5A–C). Among the three treatment groups, combination therapy induced the most profound suppression. Following seven doses, treatment was withdrawn. Notably, tumors in monotherapy groups exhibited rebound growth, while combination-treated tumors maintained sustained inhibition throughout the observation period (Fig. 5B). Notably, LBH589 monotherapy demonstrated slower rebound kinetics than OTX015 (Fig. 5B, C), though not statistically significant. The combination treatment was associated with a transient, moderate body weight loss (approximately 10%), from which mice fully recovered within 6 days after treatment cessation (Fig. 5B). Immunohistochemical analysis revealed that combined LBH589/OTX015 treatment reduced the expression of the proliferation marker Ki67 in tumor tissues (Fig. 5D, E).

**Fig. 6:**
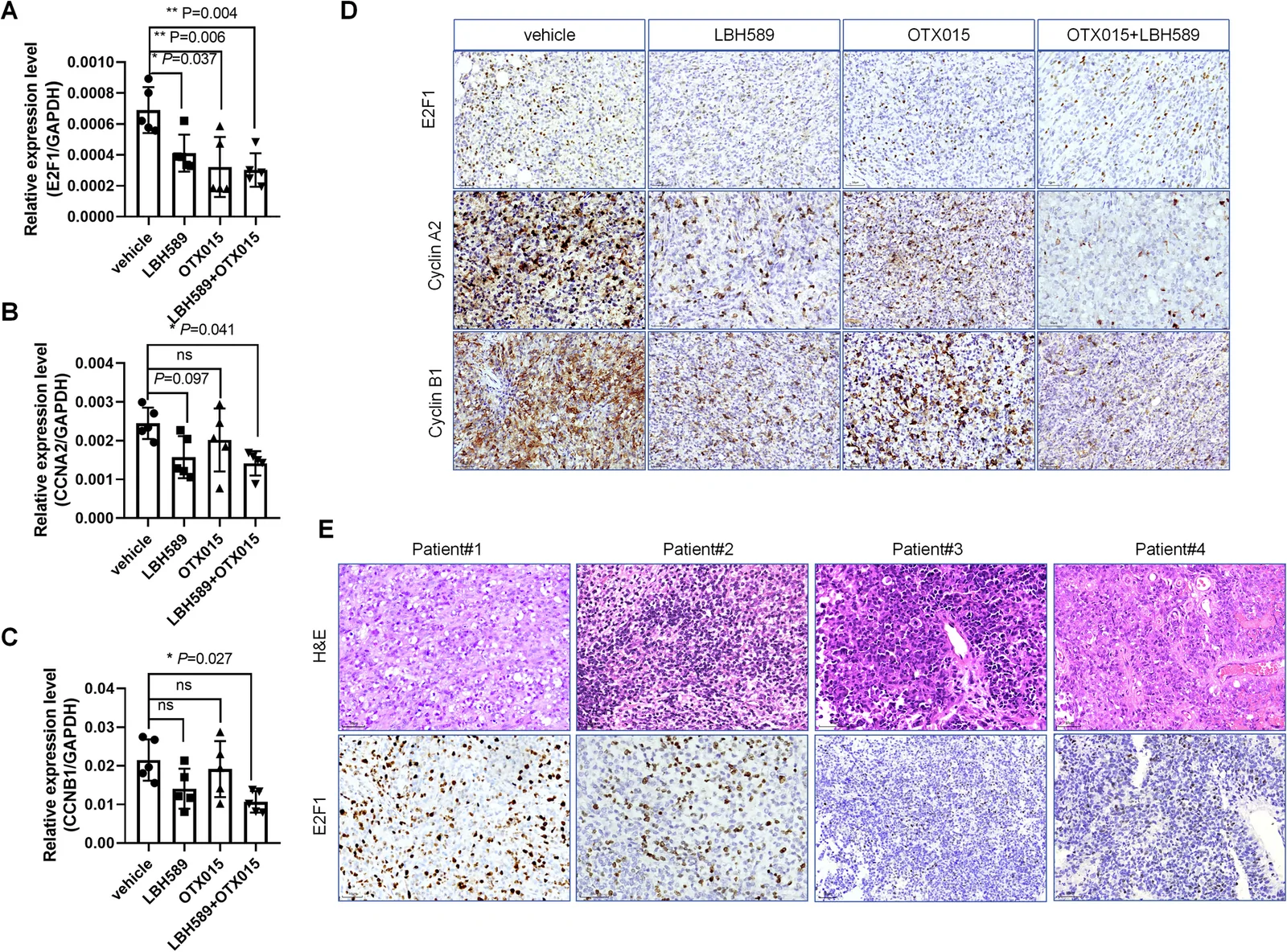
Dual HDAC and BET inhibition synergistically suppresses E2F1 in vivo. *E2F1* (**A**), *CCNA2* (**B**), and *CCNB1* (**C**) mRNA levels in A204 xenograft tumors from each treatment group, measured by RT-qPCR and normalized to *GAPDH*. **D** Representative immunohistochemistry (IHC) for E2F1, cyclin A 2 and cyclin B1 in tumor sections from the indicated groups. Scale bar, 50 µm. **E** Representative H&E staining and E2F1 IHC in primary tumor samples from MRT patients. Scale bar, 50 µm. Data are mean ± SD (*n* = 5 mice per group for **A**–**D**). Statistical significance was determined by one-way ANOVA with Tukey’s multiple comparisons test; *P < 0.05, **P < 0.01; ns not significant.

To determine whether the anti-tumor effects involve E2F1 suppression in vivo, we analyzed *E2F1* expression at the mRNA and protein levels. Marked downregulation of *E2F1* and its target genes was observed across all treatment groups (Figs. 6A–C and S7B), with the most substantial decrease in E2F1, cyclin A2 and cyclin B1 protein evident in the combination group (Figs. 6D and S7B). These data indicate that the potent in vivo anti-tumor activity of LBH589/OTX015 is mediated, at least in part, through suppression of E2F1 signaling. Consistent with prior reports [25, 26], E2F1 was aberrantly elevated in primary MRT patient samples (Fig. 6E) and several other sarcomas, such as desmoplastic small round cell tumor (DSRCT) and alveolar rhabdomyosarcoma (ARMS) (Fig. S8), underscoring the therapeutic relevance of targeting this pathway.

We further investigated the effects of LBH589 and OTX015 in a patient-derived tumor xenograft (PDX) model. To validate this model, we compared the original *SMARCB1*-deficient atypical teratoid/rhabdoid tumor (AT/RT) tissue with the generated PDX through H&E staining, INI1 (SMARCB1) immunohistochemistry, and targeted NGS sequencing of the PDX. The results verified that the PDX accurately represented the SMARCB1-deficient AT/RT (Fig. S9A, and Table S1). Subsequently, in vivo experiments were carried out according to the procedure described in Fig. S9B. Consistent with the findings in A204 cell line xenografts, the combination therapy demonstrated superior antitumor efficacy compared to LBH589 or OTX015 used alone (Fig. S9C–E).

In summary, our findings demonstrate that combined HDAC and BET inhibition effectively suppresses MRT progression in vivo and warrants further investigation as a promising therapeutic strategy for this aggressive malignancy.

## Discussion

MRT demonstrates broad anatomical tropism, with distribution patterns now well-established through three decades of clinicopathological characterization. Central nervous system manifestations—designated atypical teratoid/rhabdoid tumors (AT/RT)—comprise the predominant subtype (65%), while extracranial presentations occur primarily in the kidney (9%) and soft tissues (26%) [27]. Strikingly, comparative survival analyzes reveal significantly improved 5-year overall survival and disease-free intervals in extracranial versus AT/RT cohorts. This survival advantage likely derives from higher rates of gross total resection (GTR), greater radiotherapy (RTx) eligibility, and older age at diagnosis [28]. Nevertheless, a substantial proportion of patients still fail to achieve a cure with conventional therapies, underscoring the critical unmet need for molecularly targeted approaches beyond conventional cytotoxic regimens. In this study, we demonstrate that combined inhibition with LBH589 (panobinostat) and OTX015 (birabresib) synergistically inhibited MRT growth, supporting further evaluation of this dual epigenetic targeting approach as a potential therapeutic strategy. Mechanistically, we found that LBH589/OTX015 restores expression of p21 and p16, which are downregulated upon SMARCB1 inactivation [20]. This restoration attenuates the Cyclin D1–CDK4/6–pRb–E2F signaling axis, inhibiting Rb phosphorylation, reducing free E2F1 protein levels, and ultimately inducing G1-phase cell cycle arrest.

SMARCB1 inactivation constitutes the primary genetic driver of MRT, with minimal recurrence of other pathogenic alterations [4]. While biallelic SMARCB1 inactivation accounts for >95% of cases, rare SMARCA4 inactivation drives the remainder. Preclinical research has consequently centered on targeting SMARCB1-dependent epigenetic dysregulation—notably through HDACs, DNA methyltransferases (DNMTs), and enhancer of zeste homolog 2 (EZH2)—to counteract aberrant transcriptional silencing in MRT. Clinically, inhibitors of these epigenetic modulators—including the pan-HDAC inhibitor panobinostat (LBH589)—have advanced to trials. A phase II study evaluating LBH589 as AT/RT maintenance therapy (NCT04897880) was initiated in 2021 but closed prematurely due to drug supply limitations. Although the HDAC inhibitor vorinostat exhibits acceptable tolerability in AT/RT cohorts [29, 30]; however, a rigorous evaluation of its therapeutic efficacy is warranted. Preclinical studies in MRT have demonstrated that low-dose LBH589 induces growth arrest, intratumoral ossification, and lineage maturation of rhabdoid tumor cells both in vitro and in vivo [11]. Orthotopic xenograft data further demonstrate suppressed tumor progression and extended survival; critically, however, treatment cessation precipitates rapid tumor regrowth [31] —consistent with our observations (Fig. 5B). This inherent epigenetic plasticity underpins therapeutic resistance in MRT, underscoring the insufficiency of LBH589 monotherapy.

BET inhibitors (e.g., OTX015) exert broad anti-oncogenic activity by epigenetically repressing multiple oncogenic transcripts. Although OTX015 demonstrates dose-linear pharmacokinetics and an acceptable safety profile (ClinicalTrials.gov identifiers: NCT02698176, NCT02296476) [32], its single-agent efficacy against solid tumors remains limited. Mounting preclinical evidence supports synergistic activity between BET inhibitors and HDAC inhibitors [33]. Our results establish that LBH589 and OTX015 combination therapy elicits potent synergistic growth suppression in MRT models in vitro and in vivo. Mechanistically, this combination induces G1/S phase arrest—consistent with established effects of BET inhibitors in inducing G₁ arrest [34] and HDAC inhibitors in promoting cell cycle blockade [35]. Critically, *SMARCB1* deficiency derepresses the epigenetically silenced tumor suppressors p21 and p16—core regulators of the cyclin D1–CDK4/6-Rb axis. Pharmacologic HDAC inhibition reinstates their expression, while concurrent BET inhibition (OTX015) enhances this epigenetic restoration in MRT. Concurrently, LBH589/OTX015 combination therapy ablates E2F1 expression—a master cell cycle transcriptional regulator. Mechanistically, re-expression of p21/p16 attenuates cyclin D1–CDK4/6 kinase activity, leading to sustained hypophosphorylation of Rb. This stabilizes the Rb–E2F1 repressor complex, thereby preventing E2F1–mediated transcriptional activation of its target genes [24].

E2F1 is aberrantly expressed in MRTs, as observed in both our study and those of others. Knockdown of E2F1 in the AT/RT cell line Re1P6 induces G1 phase arrest [25], which is consistent with our observations after LBH589/OTX015 treatment. Furthermore, high E2F1 expression is correlated with poor survival in AT/RT patients across three independent cohorts [25]. Although we were unable to examine cyclin A2 or cyclin B1 expression in patient samples due to restricted access, previous studies have reported that these known E2F1 targets are aberrantly expressed in AT/RT [36], which further underscores the therapeutic relevance of targeting this pathway. Notably, since MRTs are characterized by a SMARCB1-dependent *MYC* gene expression signature, the reconstitution of SMARCB1 reorganizes the chromatin landscape around the *MYC* oncogene, affects its distal enhancers, and results in a significant reduction in MYC expression [36]. Similarly, we observed a decrease in chromatin accessibility in the distal enhancer region of MYC and a reduction in the protein level of MYC after the combined treatment with LBH589/OTX015 (Fig. S6F, G), which may contribute to cell cycle arrest. This implies that LBH589/OTX015 may regulate the cell cycle through multiple pathways.

Moreover, modulation of histone acetylation attenuates the promoter occupancy and recruitment of the transcription factor E2F1, thereby altering the expression of its downstream target genes involved in cell cycle regulation, apoptosis, and DNA repair pathways, including the *E2F1* gene itself [37, 38]. E2F1 acts as a key transcriptional switch that drives the expression of genes essential for late G1/S-phase transition and cell cycle advancement. The LBH589/OTX015 combination potently inhibits this central regulator and its associated transcriptional amplification, thereby restricting MRT cells in the G₁ phase. Mechanistically, the cyclin E promoter is repressed by an HDAC-pRB-hSWI/SNF complex that dissociates upon pRB phosphorylation, allowing transcriptional activation. In contrast, repression of the cyclin A promoter is maintained by a more stable pRB-hSWI/SNF complex. This differential regulatory stability may explain, at least in part, the observed upregulation of cyclin E1 following LBH589 treatment.

In summary, our study elucidates the inhibition of the CDK4/6-Rb-E2F axis as a critical mechanism underlying the vulnerability of MRT cells to combined HDAC and BET inhibition. Specifically, dual targeting restores expression of the tumor suppressors p21 and p16, which in turn attenuates cyclin D1–CDK4/6 kinase activity, leading to sustained Rb hypophosphorylation and consequent transcriptional silencing of E2F1-a master regulator of cell cycle progression. Our data demonstrate that co-inhibition of HDAC and BET effectively suppresses MRT growth in vitro and in vivo. These findings provide compelling evidence for further translational development of this epigenetic combination therapy as a promising therapeutic strategy for patients with MRT.

## Materials and methods

### Cell culture

A204, G401 and KYM-1 malignant rhabdoid tumor cell lines were kindly provided by Dr. Marc Ladanyi (Memorial Sloan Kettering Cancer Center). Prior to experimentation, the SMARCB1-deficient status of each cell line was validated by targeted gene sequencing (Table S1), and all cultures were confirmed to be free of mycoplasma contamination (Lonza, LT07-318). A204 cells were cultured in McCoy’s 5A (modified) Medium (Gibco, 16600082), while G401 and KYM-1 cells were grown in Dulbecco’s modified Eagle’s medium-F12 (Gibco, 12634028). All media were supplemented with 10% fetal bovine serum (FBS) (Gibco, 10099141C) and 1% penicillin/streptomycin (Gibco, 15140122). Cells were incubated at 37 °C in a humidified atmosphere containing 5% CO₂ (Thermo Fisher Scientific).

### Epigenetics compound screen

The MRT cell lines A204, G401 and KYM-1 were plated in white 96-well plates at an optimized density of 10,000 cells per well. Following overnight attachment, cells were exposed to an epigenetics compound library at a concentration of 2.5 µM. After 72 h of treatment, cell viability was assessed by adding AlamarBlue reagent (Thermo Fisher Scientific, A50101) to each well and incubating for an additional 4 h. Fluorescence was then measured using a microplate reader (Thermo Fisher Scientific, Varioskan LUX). Viability percentage was calculated by normalizing the fluorescence signal from compound-treated wells to that of vehicle-treated controls. All experiments were performed in triplicate. A complete list of the 181 compounds comprising the epigenetics compound library (Selleck, L1900) is provided in the Supplementary File (Table S2).

### Calculation of IC_50_ and combination index

The HDAC inhibitor LBH589 (Selleck, S1030) and the BET inhibitor OTX015 (Selleck, S7360) were dissolved in DMSO according to the manufacturer’s protocol. MRT cells were treated with LBH589 or OTX015 at different concentrations for 72 h, with an equal volume of DMSO (Sigma, D2650) serving as the vehicle control. All treatments were performed in triplicate. IC_50_ values were calculated using GraphPad Prism (V8) by normalizing raw fluorescence readings and fitting the data to a four-parameter dose–response model via nonlinear regression. Drug synergy was assessed using the Chou–Talalay method implemented in CompuSyn software. The CI was calculated based on monotherapy and combination dose–response data, where CI = 1 indicates an additive effect, CI < 1 indicates synergy, and CI > 1 indicates antagonism.

### RNA-seq analysis

Preliminary time-course experiments across 0, 8, 16, 24 and 48 h, monitoring key pharmacodynamic markers, histone acetylation for LBH589 and cell viability for the combination of LBH589 and OTX015. The 16-h time point meets the principles of optimal time point selection [39], it exhibited sustained target modulation, early apoptotic signaling without major viability loss (>80% viable cells), and high RNA integrity (RIN > 8). Cells were treated with LBH589, OTX015 or their combination for 16 h, and total RNA was extracted by RNAzol (Molecular Research Center, RN190) followed by purification with the Direct-zol RNA MiniPrep Kit (ZYMO, R2052). RNA sequencing library preparation, sequencing and primary bioinformatic analyzes were performed by BioWavelet Ltd (Chongqing, China). Briefly, libraries were constructed using EASY-RNAseq method and sequenced on Illumina HiSeq platform using the 150-bp pair-end configuration. Raw reads were processed with Trimmomatic to remove adapters and low-quality bases, then aligned to the human reference genome (GRCh38.p7). Only reads assigned to a unique genomic locus were counted using FeatureCounts, and the resulting read counts were converted to FPKM values to measure gene expression. GSEA was performed using GSEA software (Broad Institute), and hierarchical clustering based on average linkage and Euclidean distance was carried out with Multiexperiment Viewer (version 4.9, http://www.tm4.org). All data are accessible in NODE [40] (https://www.biosino.org/node) with the accession number OEP00006969 or through the URL: https://www.biosino.org/node/project/detail/OEP00006969.

### Cell cycle assay

For cell cycle analysis, cells treated with OTX015 and/or LBH589 and DMSO for 48 h were washed thrice with PBS and fixed overnight at 4 °C in 75% ethanol. Fixed cells were then pelleted by centrifugation, washed three times with PBS, and resuspended in precooled PBS. Subsequently, cells were stained in the dark for 30 min with propidium iodid (PI; BD Biosciences, 556547) and RNase A (Thermo Fisher Scientific, EN0531). Cell cycle distribution was analyzed using an LSRFortessa flow cytometer (BD Biosciences), and data were processed with FlowJo software.

### Quantitative real-time PCR

A204 cells were treated with LBH589 at a concentration of 35 nM and OTX015 at 350 nM; G401 cells were treated with LBH589 at 70 nM and OTX015 at 1.6 μM. All these concentrations were approximately onefold IC_50_, which were selected as representative doses for mechanistic studies. In dose–response experiments, OTX015 was used at 0.5-fold, 1-fold, and 2-fold IC_50_ in both cell lines. For LBH589, due to excessive cytotoxicity at twofold IC_50_ in G401 cells, the doses in G401 were reduced to 0.25-fold, 0.5-fold, and 1-fold IC_50_. RNA concentration was quantified using a spectrophotometer (Thermo Fisher Scientific). cDNA was synthesized using the RevertAid First Strand cDNA Synthesis Kit (Thermo Fisher Scientific, K1622). Quantitative PCR was carried out with PowerUp™ SYBR™ Green Master Mix (Thermo Fisher Scientific, A25742) according to the manufacturer’s instructions. *GAPDH* was used as the endogenous control for normalization. Relative gene expression was calculated using the 2^−ΔΔCt^ method. The primer sequences are available in the Supplementary File (Table S3).

### Capture of nascent RNAs

To capture nascent RNAs, 0.2 mM EU was incorporated into the cells for 3 h. EU-labeled RNAs were biotinylated and captured by using the Click-iT Nascent RNA Capture Kit (Thermo, C10365) in accordance with the manufacturer’s instructions.

### Western blot analysis

Western blotting analyzes were performed with whole cell lysates according to previously published procedures [41]. The following antibodies were used: anti-GAPDH (5174), anti- CDK4 (12790), anti-CDK6 (3136), anti-cyclin D1 (55506), anti-cyclin E1 (4129), anti-cyclin B1 (12231), anti-cyclin A2 (67955), anti-p21 (2947), anti-p27 (3698), anti-p16 (80772), anti-Rb (9309), anti-Phospho-Rb (Ser807/811) (8516), all from Cell Signaling Technology; anti-E2F1 (sc-251) was from Santa Cruz Biotechnology. The immunoblots were imaged and analyzed using Image Studio (LI-COR Biosciences).

### Immunohistochemistry

Tumor tissues were fixed in 4% paraformaldehyde, paraffin-embedded, sectioned, and mounted onto slides. Following deparaffinization and rehydration, tissue sections underwent antigen retrieval and blocking, then were incubated with primary antibodies against E2F1 (Santa Cruz, sc-251; 1:75), cyclin A2 (Proteintech, 18202-1-AP,1:400), cyclin B1 (Proteintech, 28603-1-AP, 1:200) or Ki67 (Cell Signaling Technology, 9449; 1:800) for 45 min at room temperature. After washing, sections were incubated with an HRP-conjugated secondary antibody for 30 min, developed with DAB, counterstained with hematoxylin, and coverslipped with gelatin mounting medium. Hematoxylin and eosin (H&E) staining was performed using a commercial kit (Abcam, ab245880) according to the manufacturer’s protocol. Categorization of immunostaining intensity was performed manually by two independent observers blinded to the treatment conditions. For each xenograft tumor sample, five randomly selected, non-overlapping fields were captured at 400× magnification. In every field, at least 400 cells were counted, yielding a minimum of 2000 total cells/case. Staining intensity was assigned a score using a semiquantitative four-category grading system: 0, no staining; 1, weak staining (light yellow); 2, moderate staining (between 1 and 3); and 3, strong staining (brown-yellow). The percentage of positive nuclei was calculated for each field, and the final score for each sample represents the mean percentage across all analyzed fields. Nuclear positivity for Ki67 was defined as clear yellow/brown staining above background, regardless of intensity. Staining score of E2F1, cyclin A2 and cyclin B1 was determined according to the staining depth and the ratio of positive stained positive cells. Staining score = staining depth × positive-stained cell ratio.

### Xenograft model

All animal experiments were conducted using 6-week-old female immunodeficient NSG mice (*NOD-Prkdc*^*scid*^*Il2rg*^*em1/Smoc*^, NM-NSG-001) purchased from Shanghai Model Organisms (Shanghai, China). Mice were subcutaneously injected with 1 × 10⁶ A204 cells into the left flank. Tumor growth was monitored by caliper measurement, and volume was calculated using the formula *V* = (*a*² × *b*)/2, where *a* is the shortest and *b* the longest diameter. The investigators responsible for body weight measurement and tumor volume measurement were unaware of the group allocation. When tumors reached approximately 50 mm³, mice were randomly assigned to four groups (*n* = 5 per group) using a computer-generated random number sequence: vehicle control, LBH589 (5 mg/kg, by intraperitoneally injection, every 2 days), OTX015 (50 mg/kg, by oral gavage, every 2 days), and the combination of LBH589 plus OTX015 at the same doses. Treatments were administered for a total of seven doses. Approximately 3 weeks after treatment initiation, mice were euthanized by cervical dislocation, tumors were excised, and samples were processed for subsequent analyzes. All procedures were approved by the Institutional Animal Care and Use Committee of Renji Hospital, Shanghai Jiao Tong University School of Medicine (Approval No. RJ2022-1031).

### Clinical samples

Ethical approval for the utilization of FFPE samples from MRT patients has been obtained from the Ethics Committee of Renji Hospital, affiliated with Shanghai Jiao Tong University School of Medicine (Approval No. LY2024-264-B), informed consent was obtained from all subjects. The clinicopathological characteristics of patients are listed in Supplementary Table 4.

### Statistical analyses

All experiments were performed in at least three independent biological replicates. All samples/animals that were successfully treated and completed the experimental protocol were included in the analysis. Data are presented as mean ± SD. Normality was checked by Shapiro–Wilk test and variance homogeneity by Brown–Forsythe test (median-based, robust to non-normality and small samples). For two-group comparisons: Mann–Whitney U test, or two-sided *t*-test if normality and homoscedasticity were met. For comparisons of three or more independent groups: Kruskal–Wallis test with Dunn’s multiple comparisons test, or One-way ANOVA with Tukey’s multiple comparisons test if parametric assumptions were satisfied. *P* < 0.05 was considered statistically significant.

## Supplementary information

## Data Availability

The datasets used and/or analyzed during this study are available from the corresponding author on reasonable request.
