# Supplementary material for: Combined inhibition of BETs and HDACs as a potential epigenetics-based therapy for malignant rhabdoid tumor
Source: Cell Death Dis. 2026 Jun 22;17(1):771. doi: 10.1038/s41419-026-09026-z (PMC13538387; doi:10.1038/s41419-026-09026-z)

Figure 3F E2F1 CCNB1 CCNE1 GAPDH

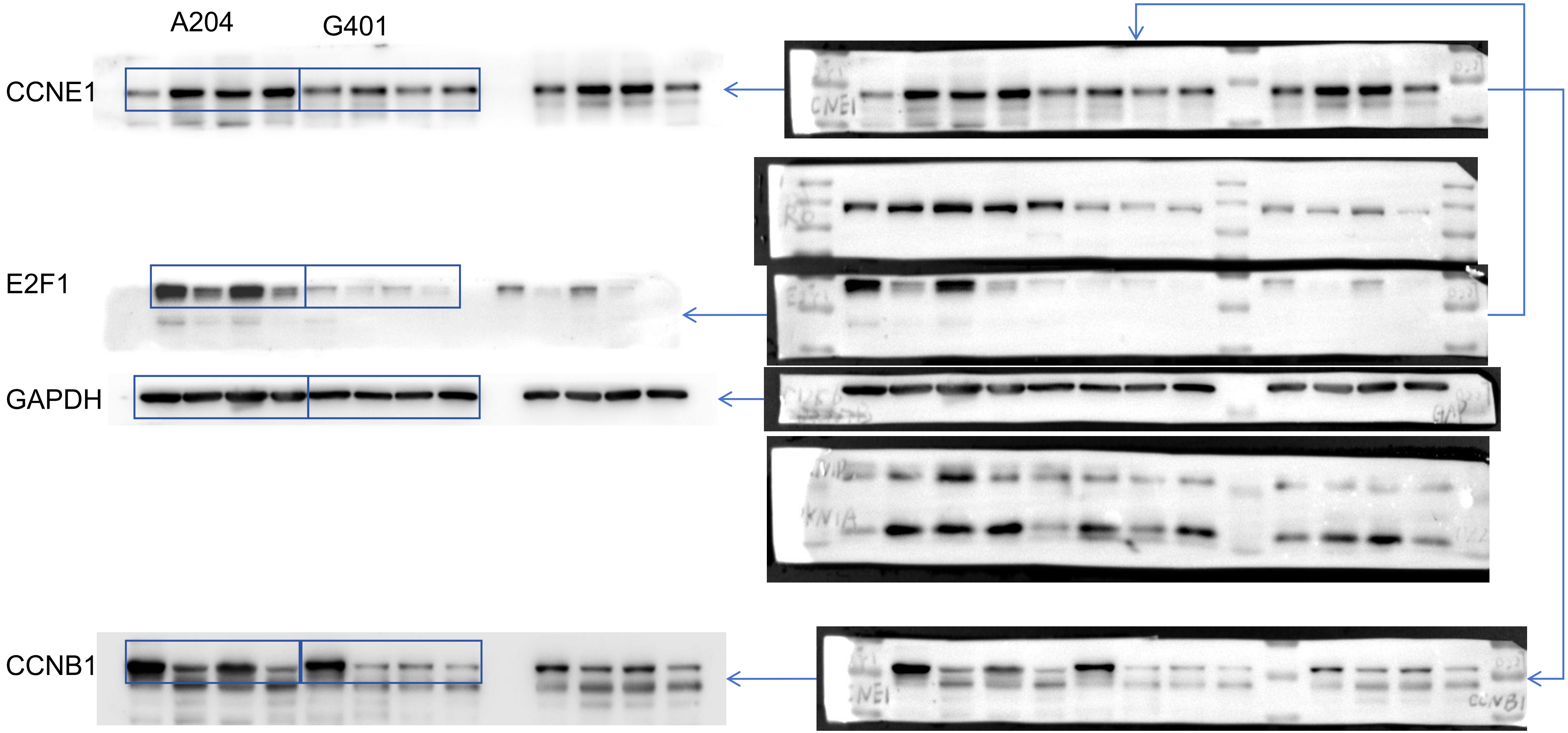

Figure 3F CCNA2 GAPDH

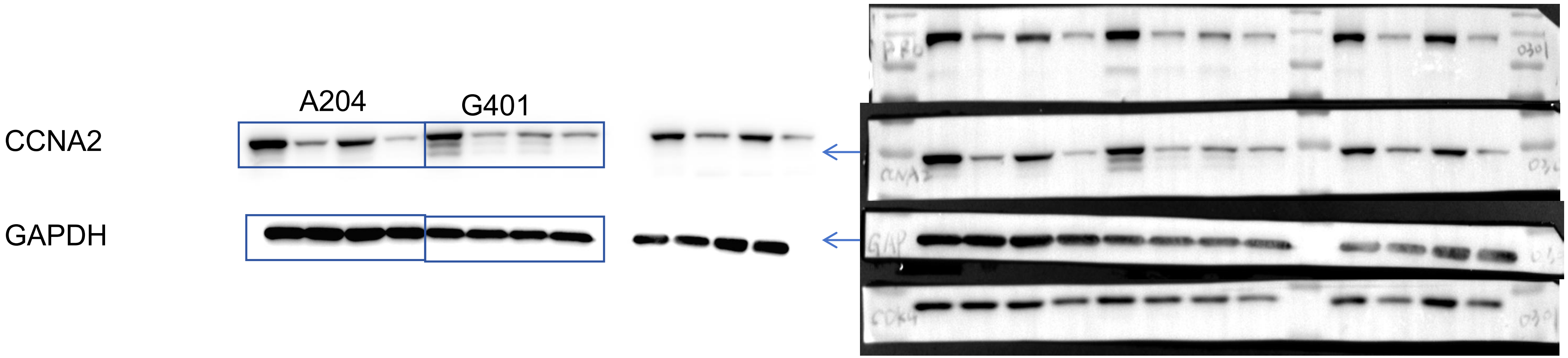

Figure 3G A204 E2F1 CCNA2 CCNB1 GAPDH

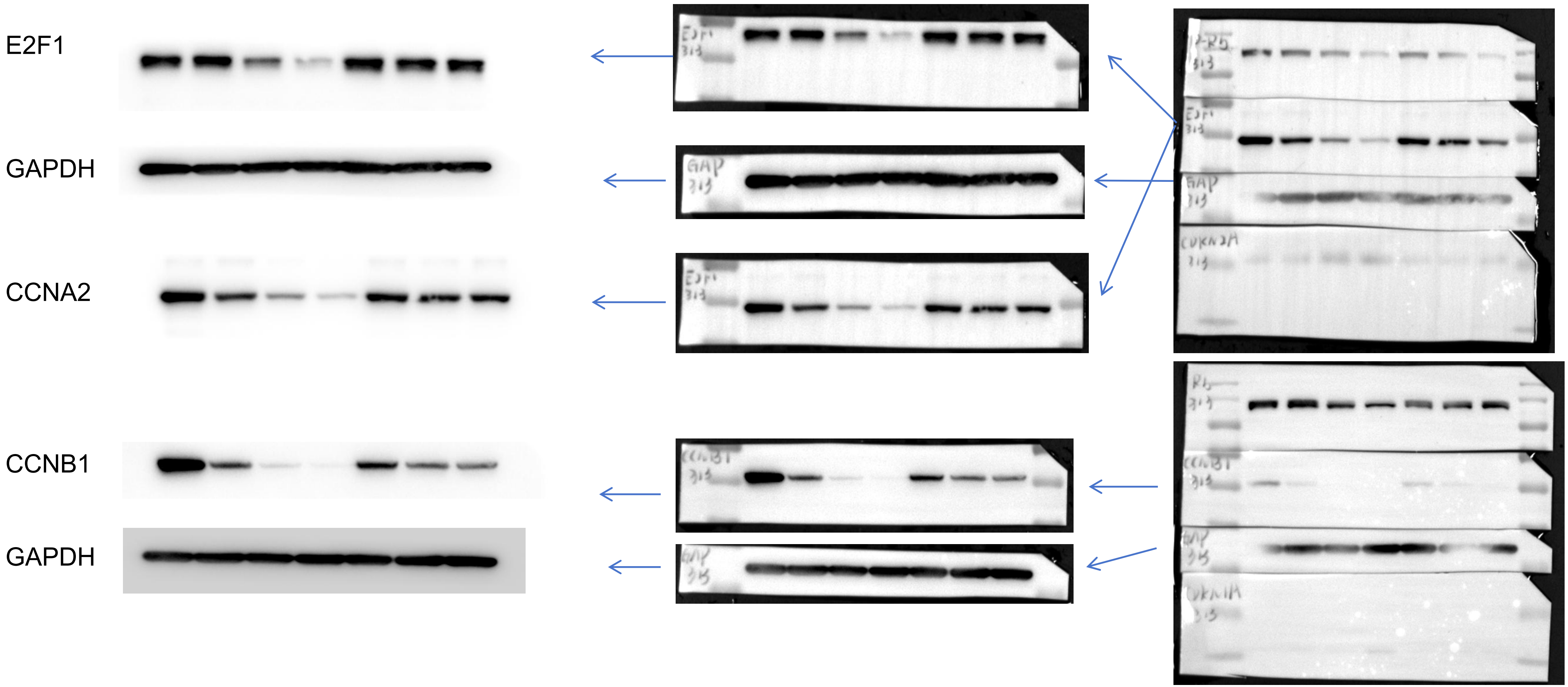

Figure 3G A204 CCNE1 GAPDH

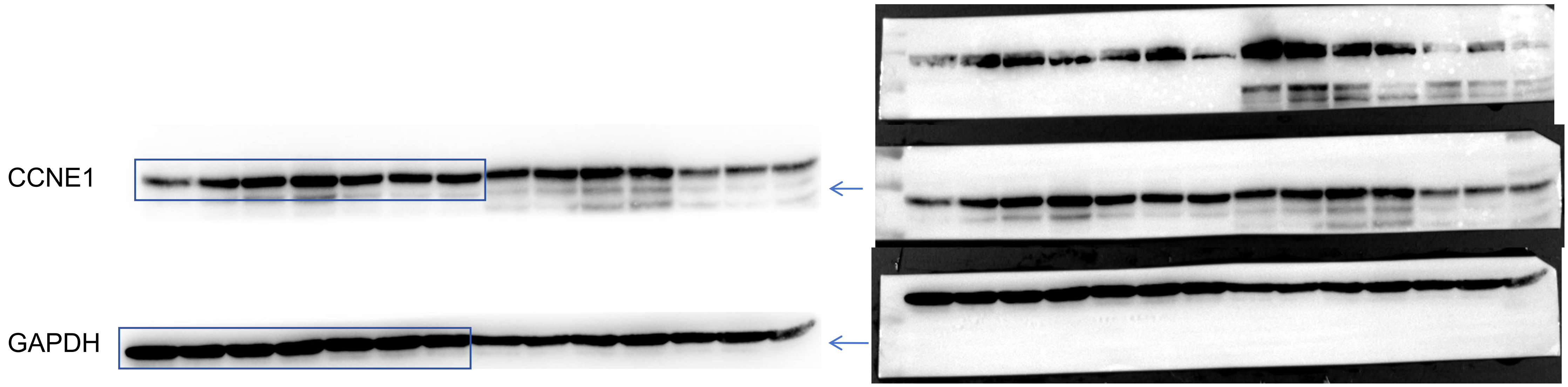

Figure 3G G401 E2F1 CCNE1 GAPDH

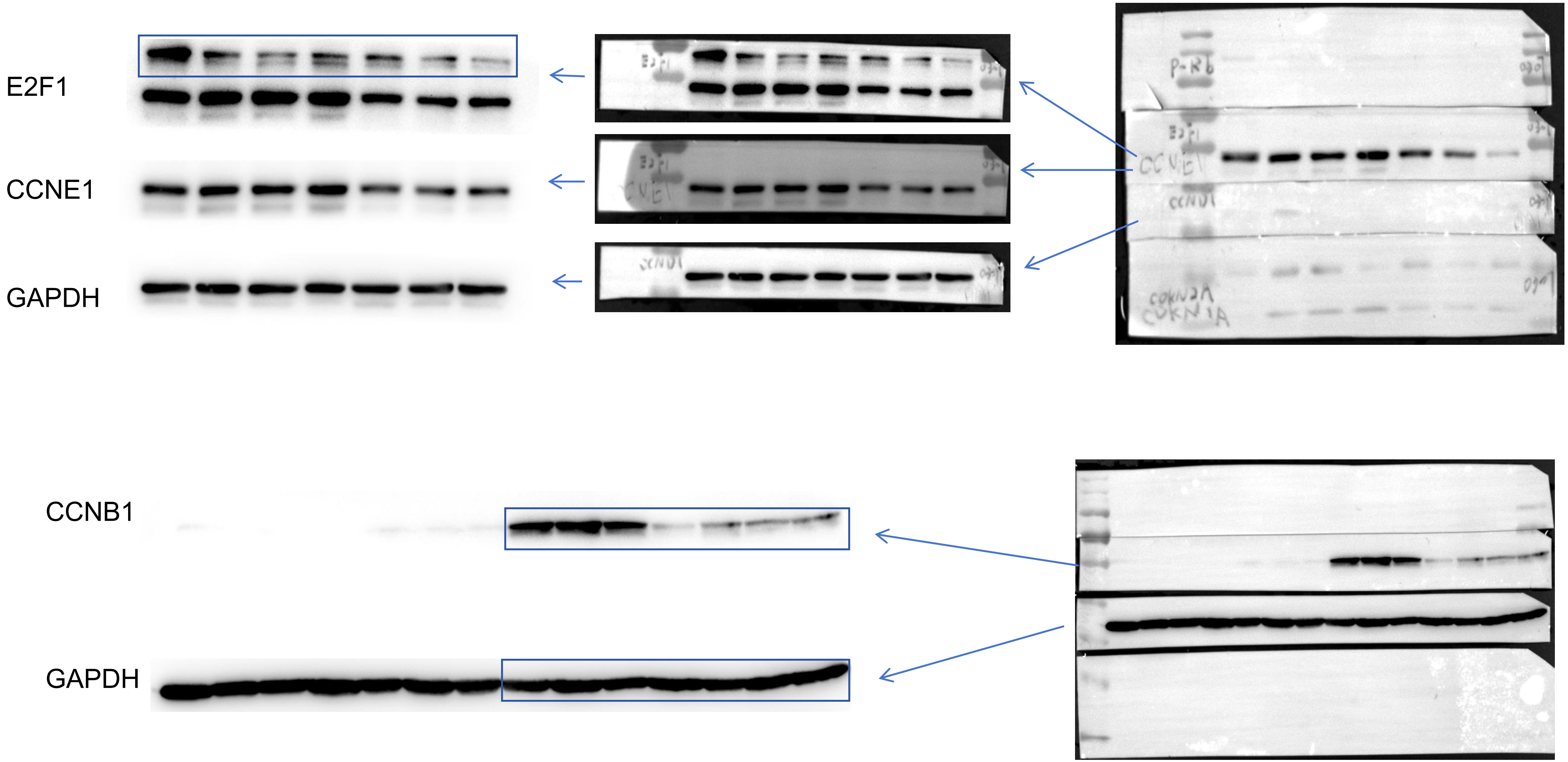

Figure 3G G401 CCNA2 GAPDH

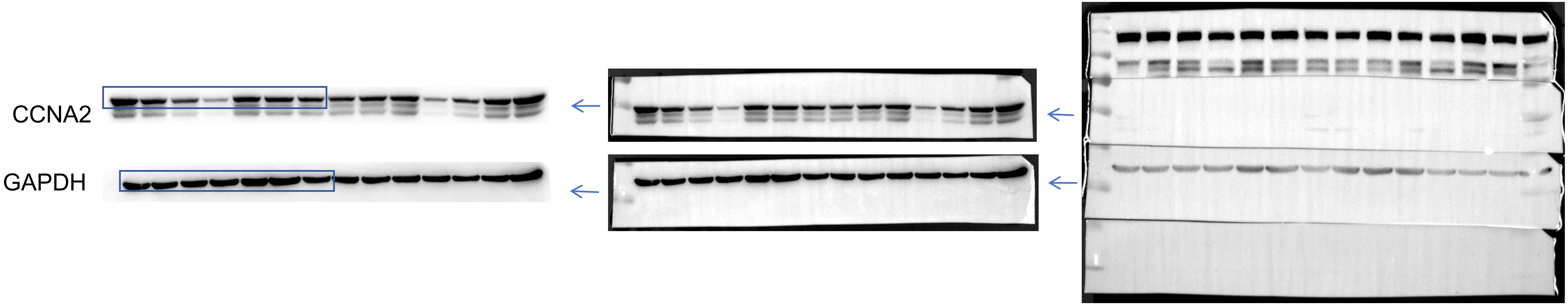

Figure 4A,B Rb CDKN1A CDK6 GAPDH

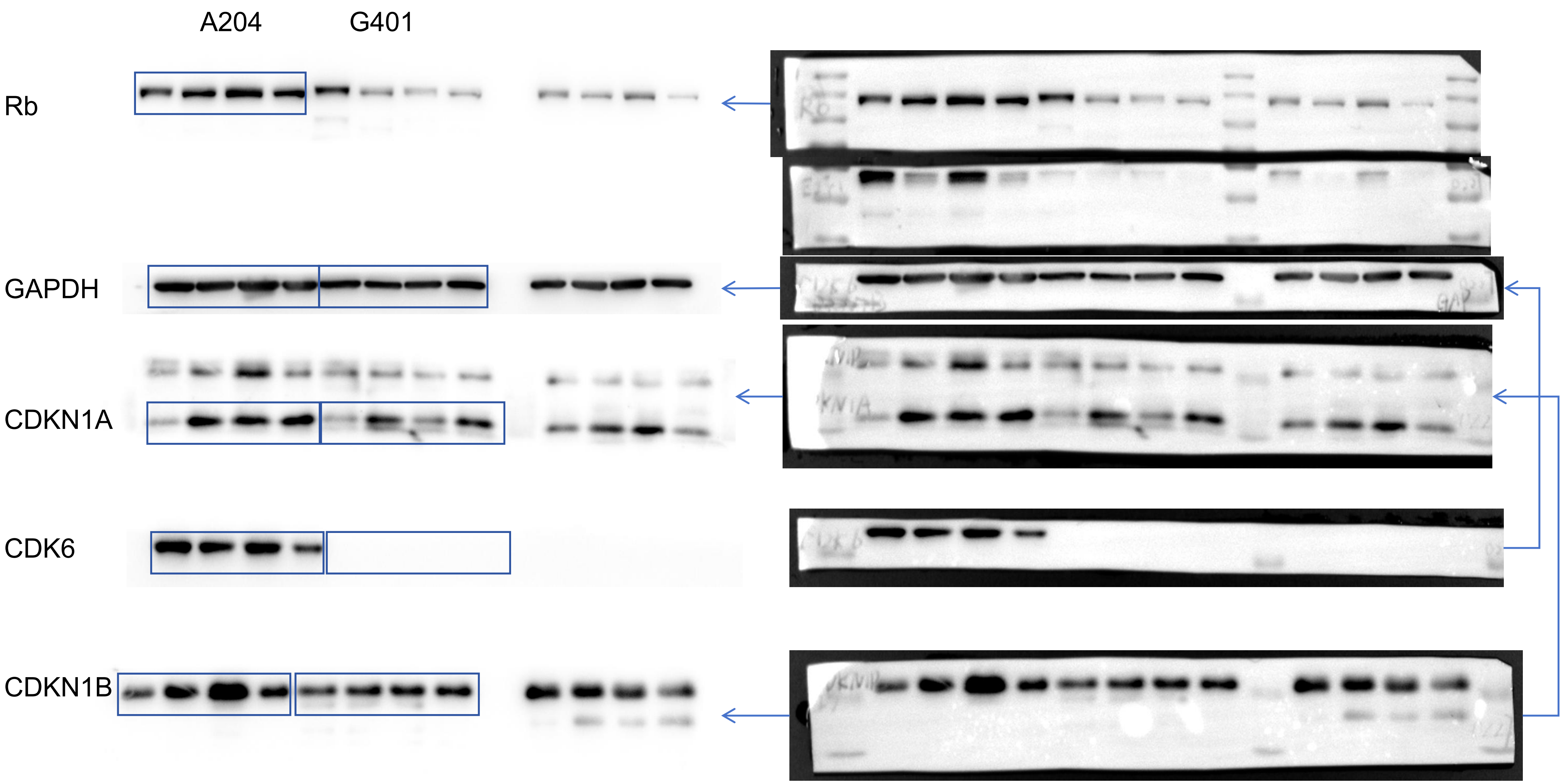

Figure 4A,B CDK4 GAPDH

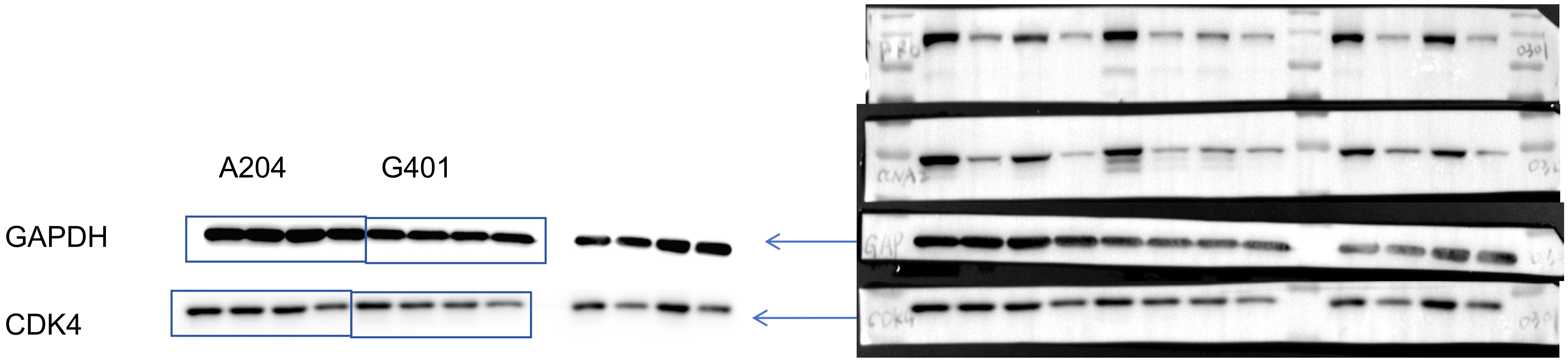

Figure 4A,B CCND1 CDKN2A GAPDH

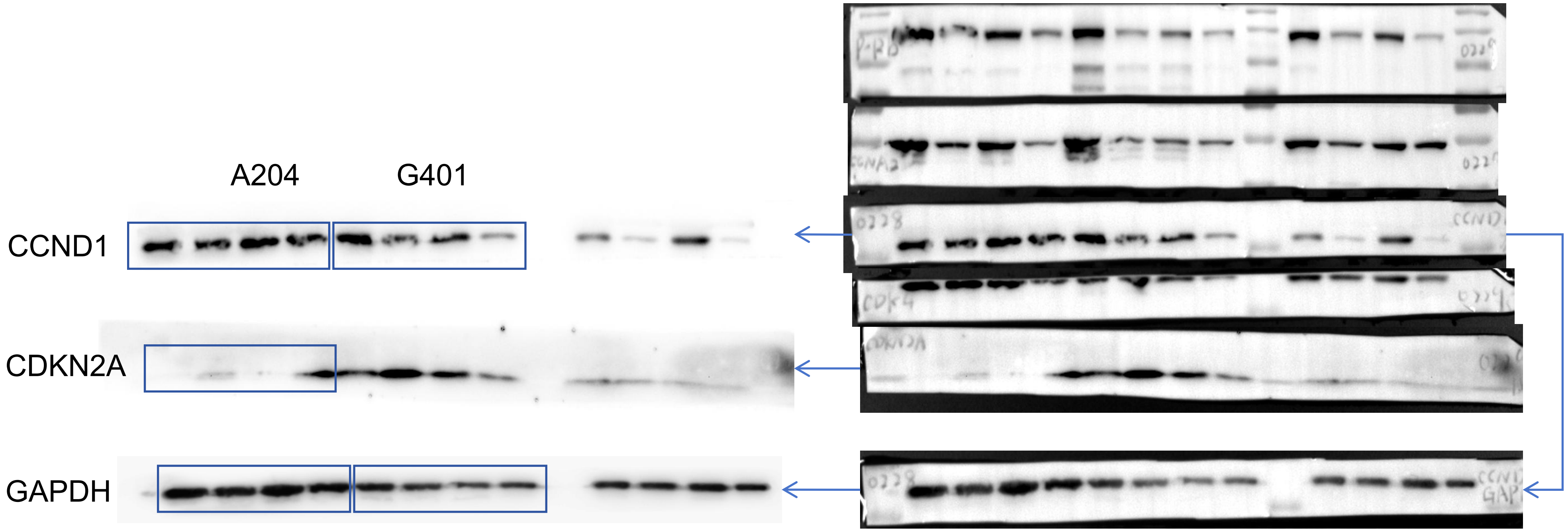

Figure 4A,B p-Rb GAPDH

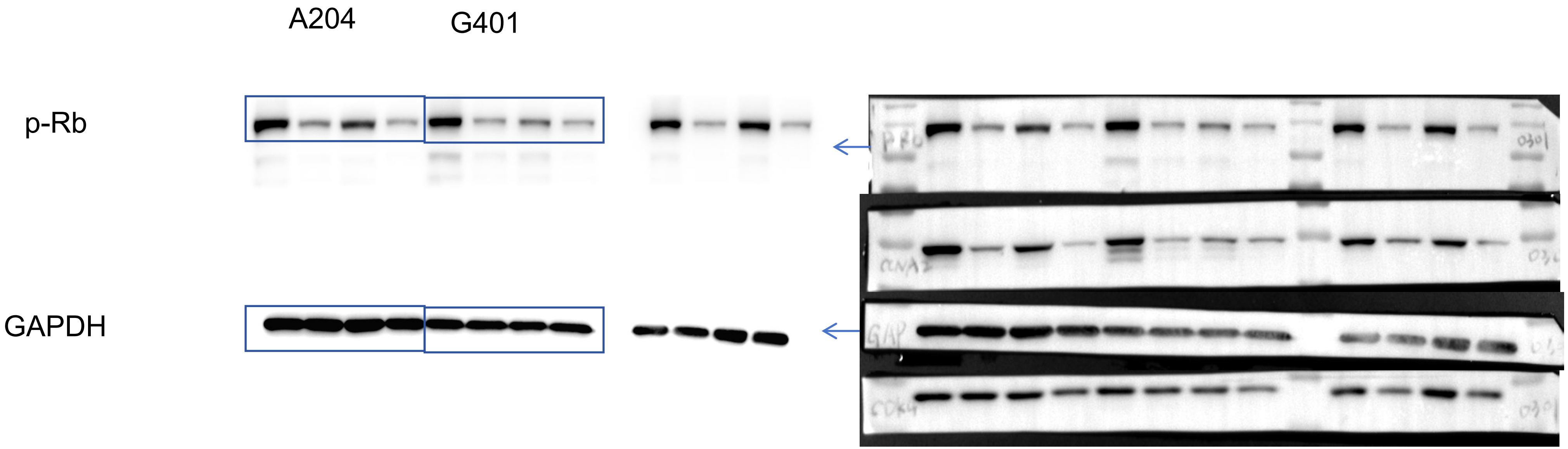

Figure 4B Rb GAPDH

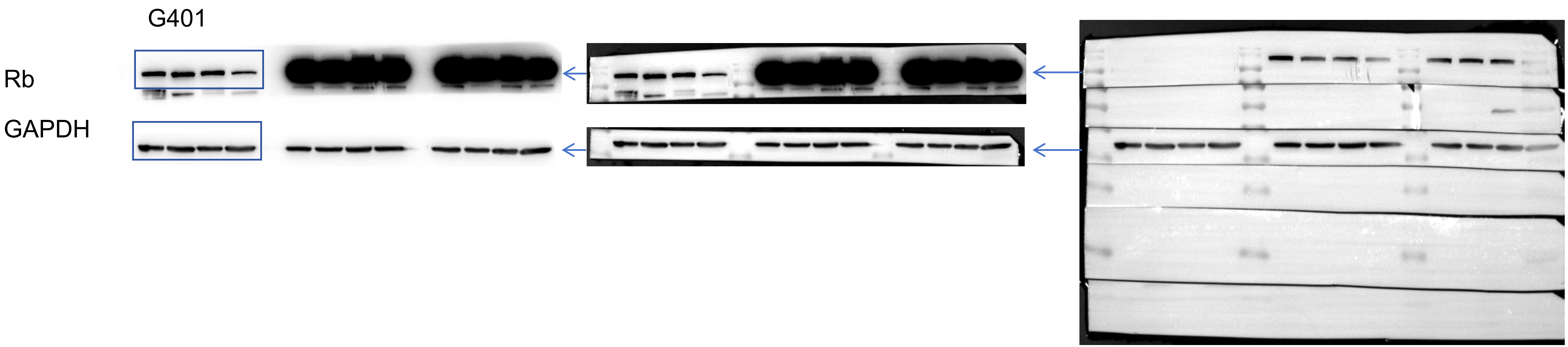

Figure 4B CDK6 CDKN2A GAPDH

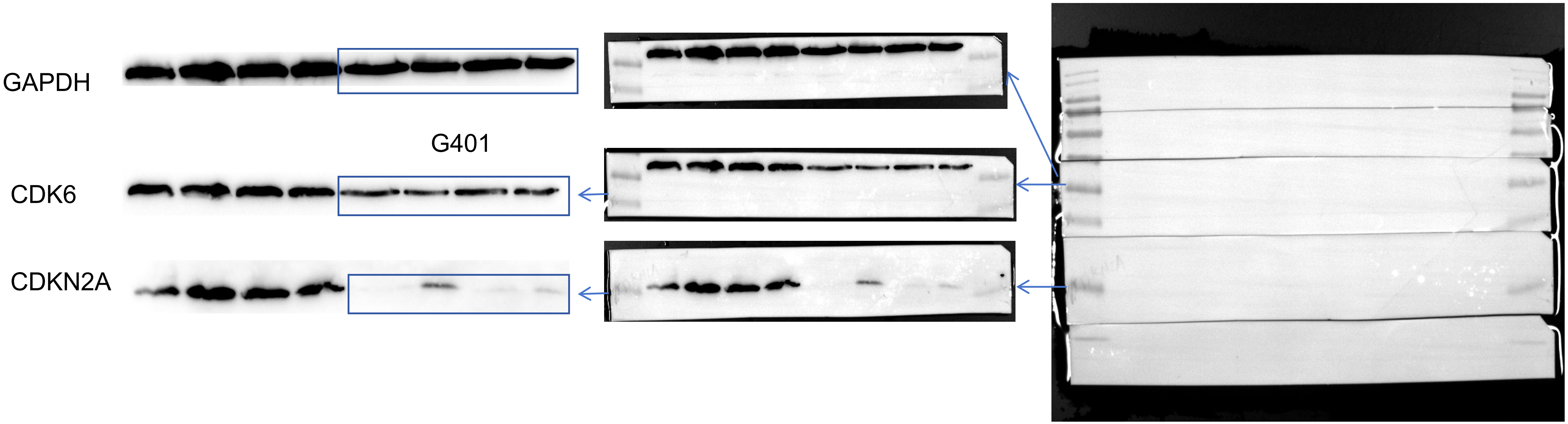

Figure 4C CCND1 GAPDH

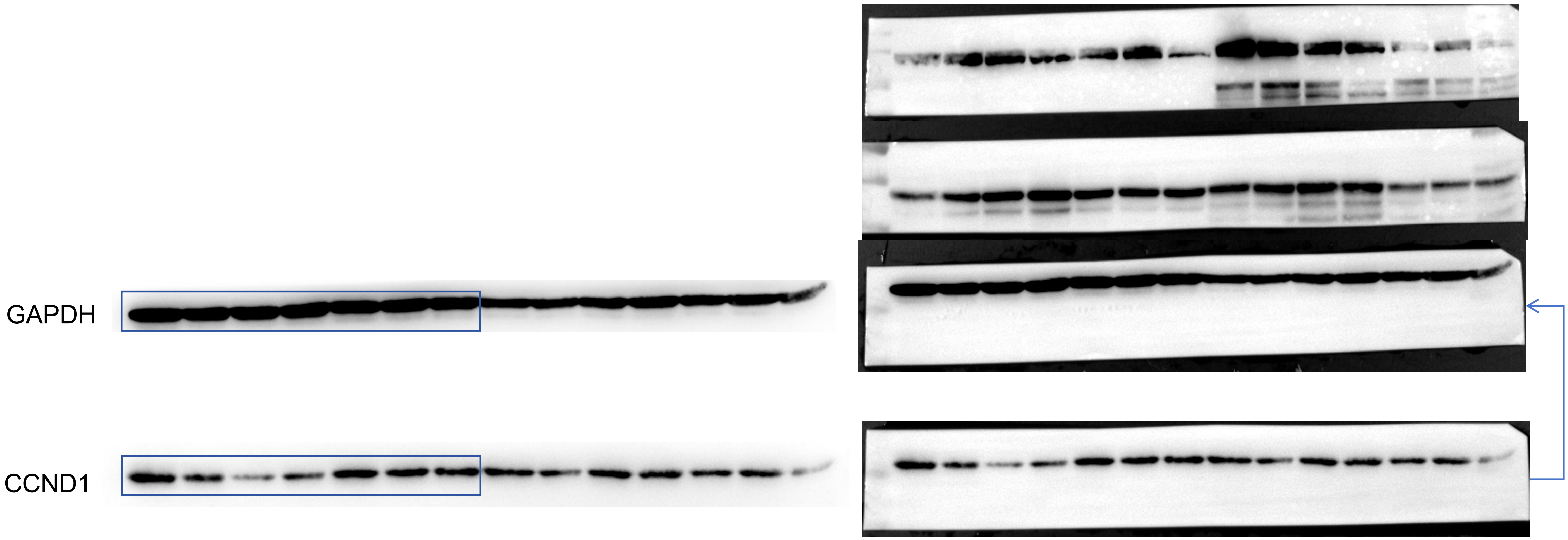

Figure 4C p-Rb CDKN1B CDKN2A GAPDH

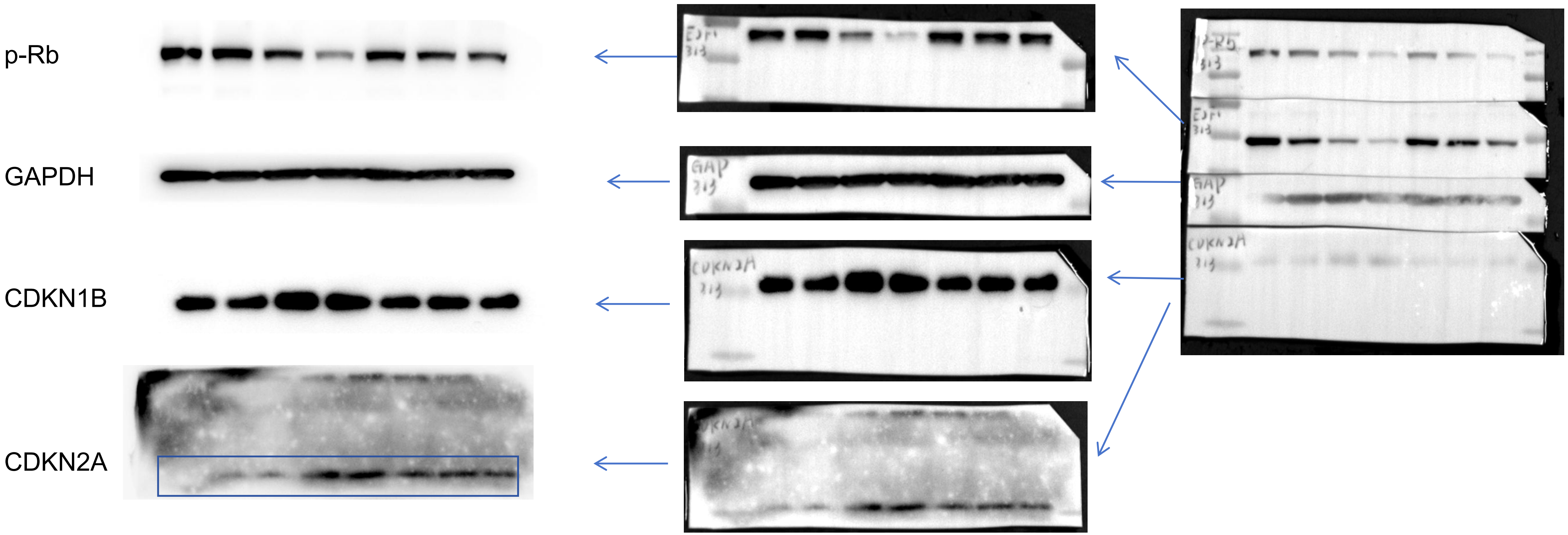

Figure 4C Rb CDKN1A GAPDH

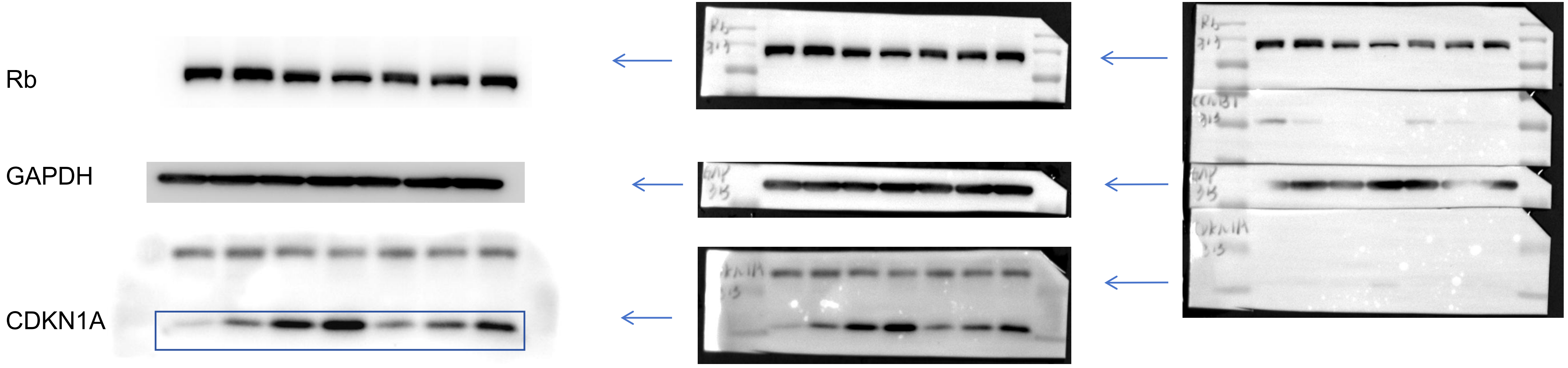

Figure 4D Rb p-Rb CCND1 CDKN1A CDKN1B GAPDH

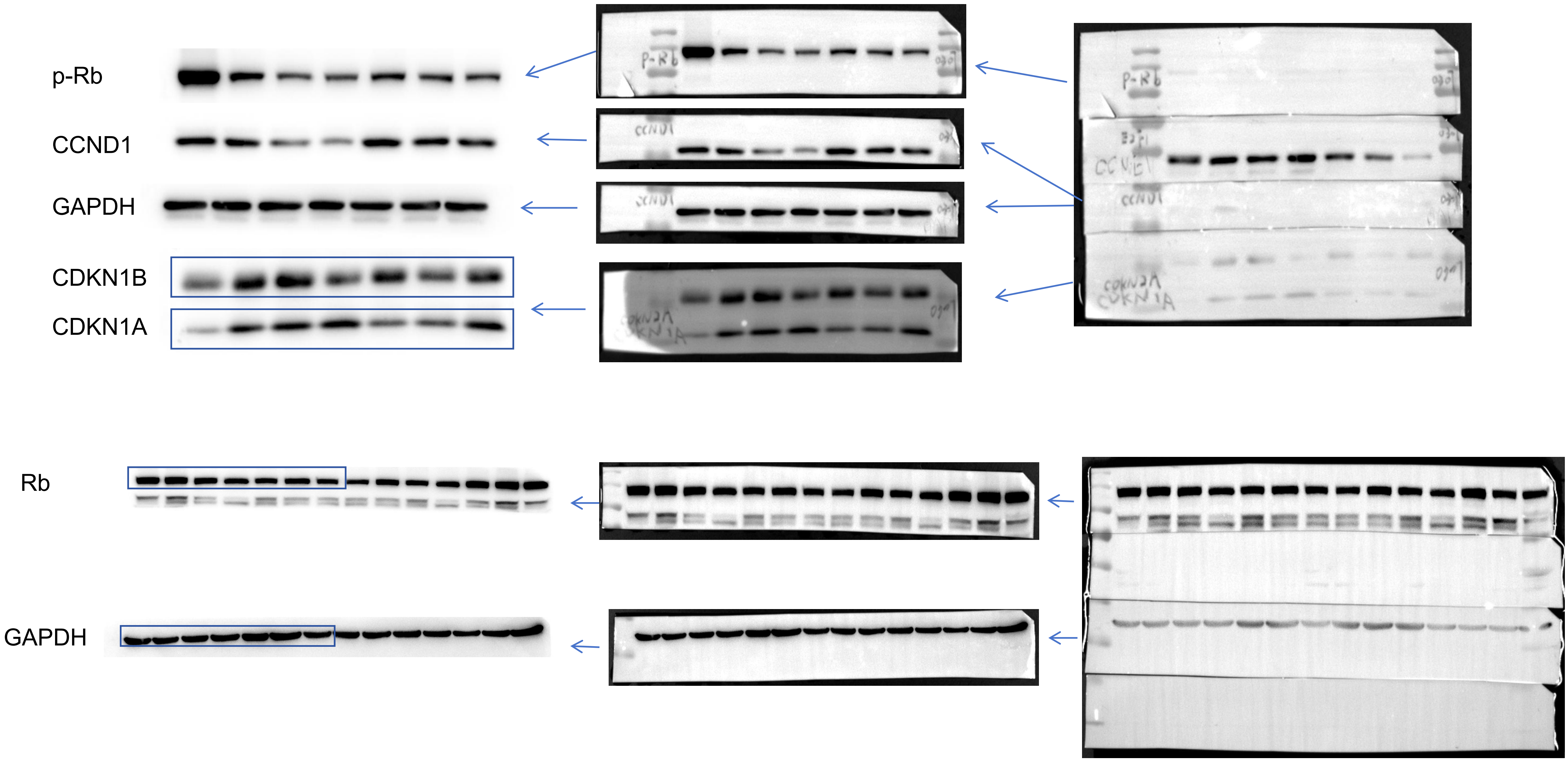

Figure S3 PARP1 cleaved-caspase3 GAPDH

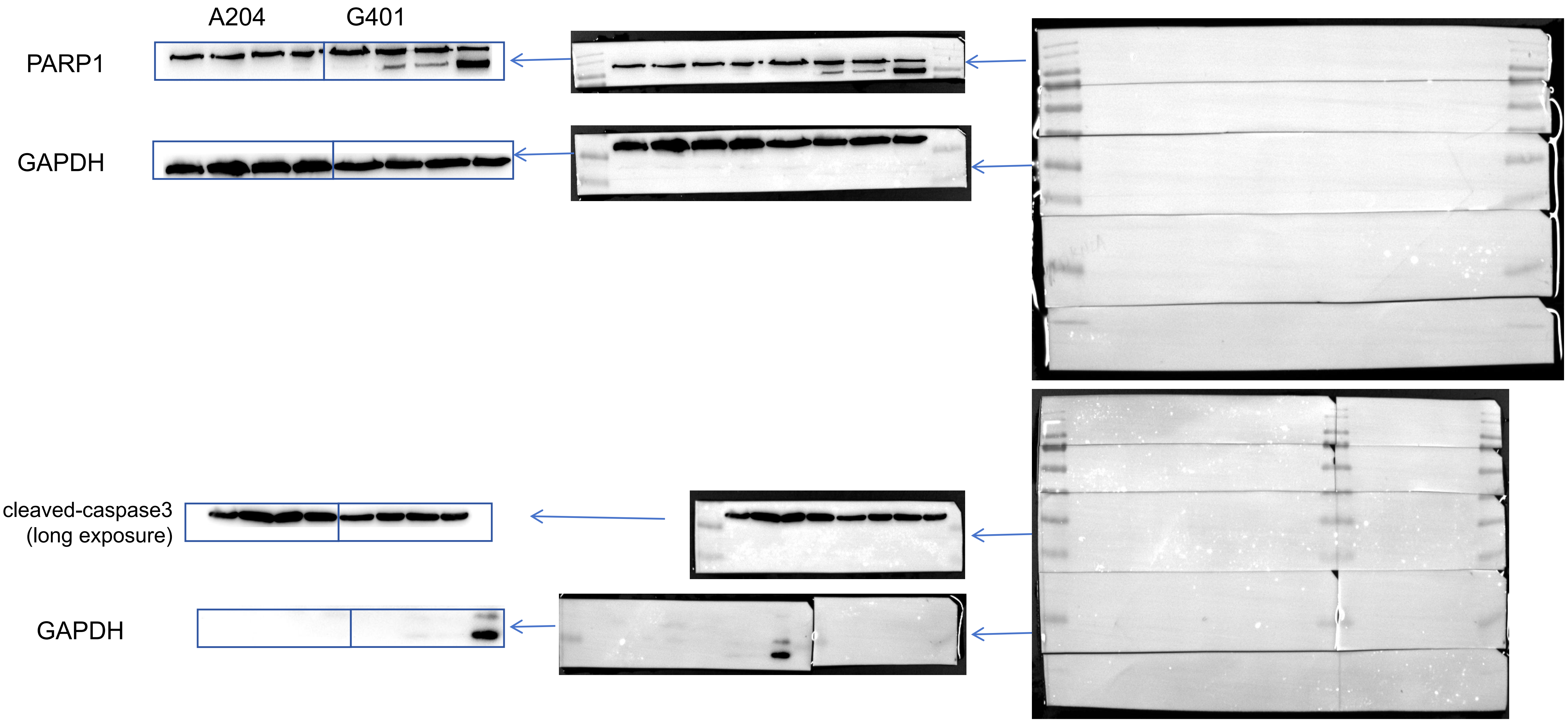

Figure S3 caspase3 GAPDH

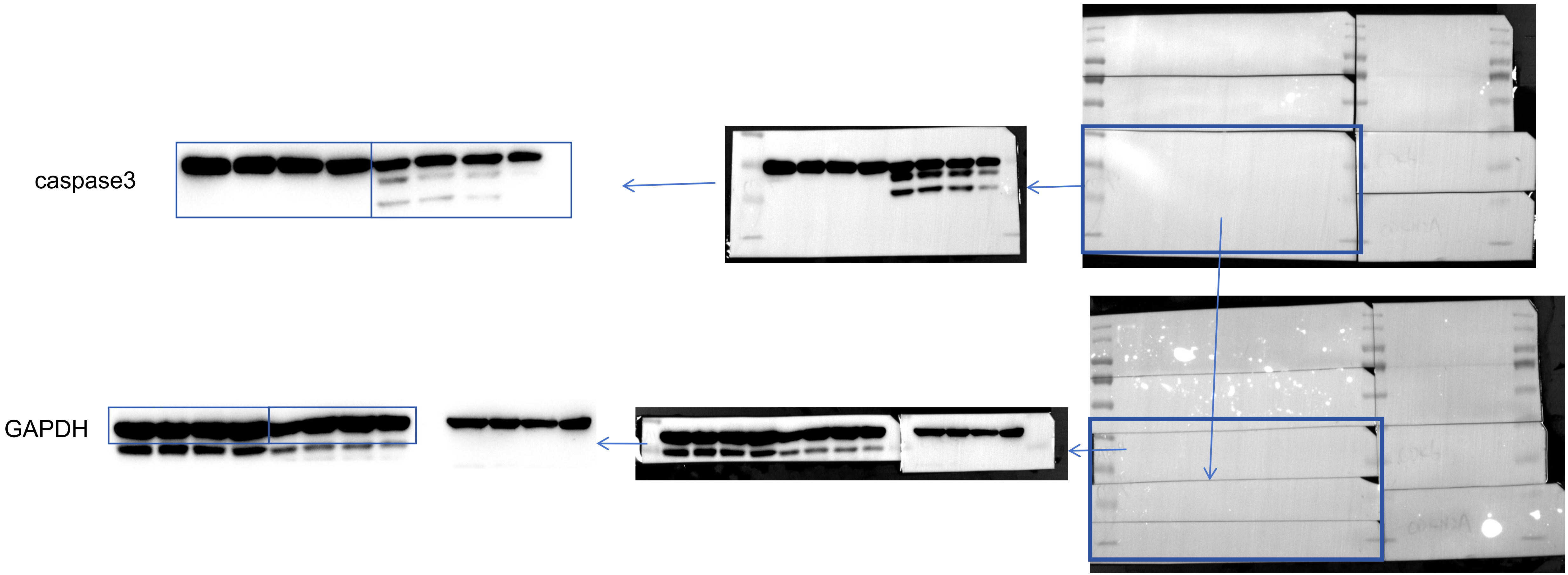

Figure S4A Acetyl-H3 Acetyl-H4 GAPDH

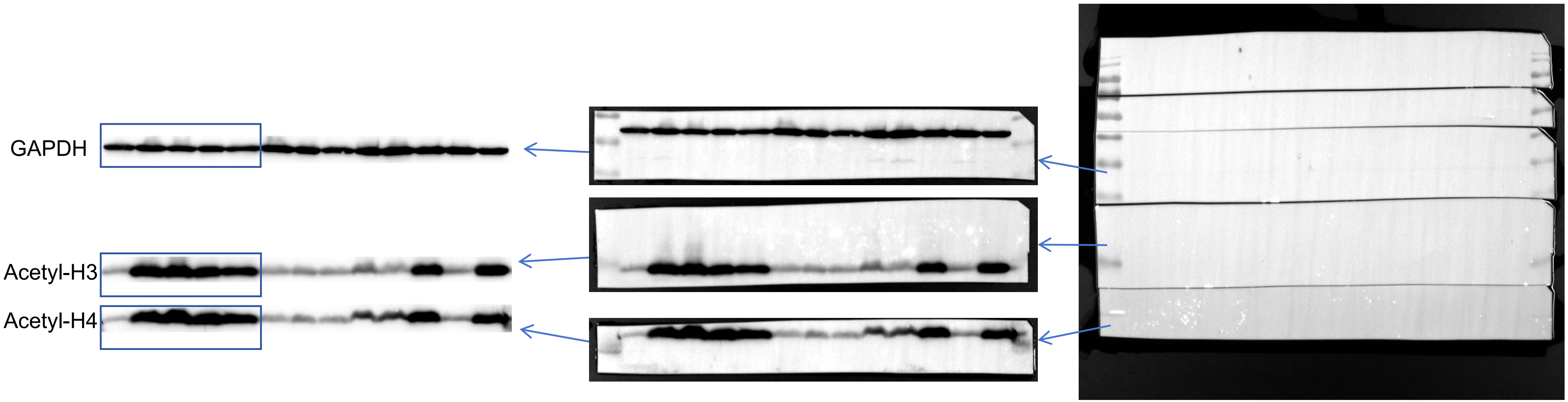

Figure S4B E2F1 CCNA2 GAPDH

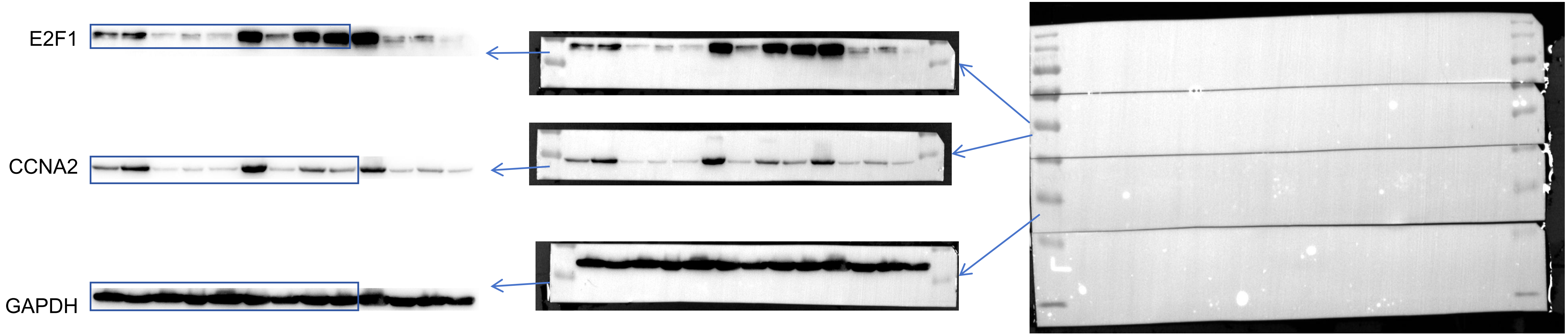

Figure S6G MYC GAPDH

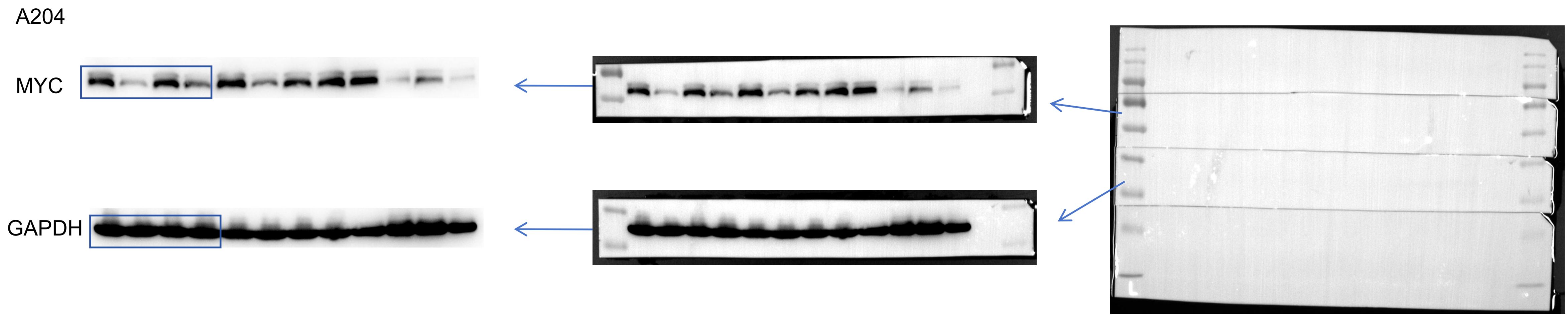

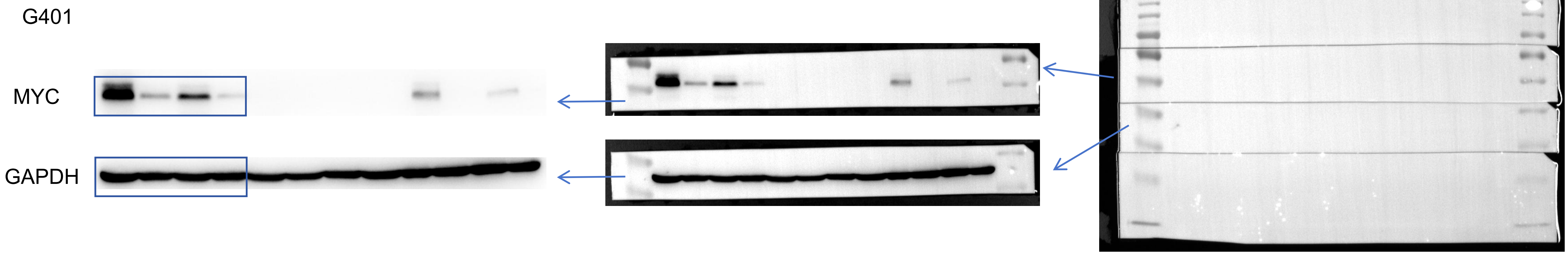

Supplement: Supplementary file 1 [file 41419_2026_9026_MOESM12_ESM.pdf]
