# Supplementary material for: Combined inhibition of BETs and HDACs as a potential epigenetics-based therapy for malignant rhabdoid tumor
Source: Cell Death Dis. 2026 Jun 22;17(1):771. doi: 10.1038/s41419-026-09026-z (PMC13538387; doi:10.1038/s41419-026-09026-z)
Supplement: Supplementary file 2 [file 41419_2026_9026_MOESM1_ESM.docx]

**Supplementary methods**

**Targeted gene sequencing**

Genomic DNA was extracted from either cell line lysates or five 10-μm sections of formalin-fixed paraffin-embedded (FFPE) tissue using the DNeasy Blood & Tissue Kit (QIAGEN, 69504) or QIAamp DNA FFPE Kit (QIAGEN, 56404), respectively, according to the manufacturer’s protocols. DNA quality was evaluated by spectrophotometry, with optical density (OD) measured at 230, 260, and 280 nm. DNA concentration was quantified using Qubit 4.0 software. Library preparation was performed as previously described [1]. Target enrichment was carried out by hybridization capture using the Sarcopact™ tumor gene panel (481 cancer-relevant genes, Geneseeq Technology Inc.) with the xGen Lockdown Hybridization and Wash Reagents Kit (Integrated DNA Technologies). Captured libraries were bound to Dynabeads M-270 (Life Technologies), amplified with KAPA HiFi HotStart ReadyMix (KAPA Biosystems), and quantified by qPCR using the KAPA Library Quantification kit (KAPA Biosystems). Sequencing was performed on the Nextseq550Dx platform (Illumina) with 2 × 150 bp paired-end reads. Raw sequencing data were demultiplexed using bcl2fastq (v2.19) and trimmed with Trimmomatic. Reads containing Ns or with low quality (quality score < 15) were removed, and the remaining reads were aligned to the human reference genome (hg19) using Burrows–Wheeler Aligner. PCR duplicates were removed with Picard (https://broadinstitute.github.io/picard/). Local realignment around indels and base quality recalibration were performed using the Genome Analysis Toolkit (GATK). Variant calling for single-nucleotide polymorphisms (SNPs) and indels was conducted with HaplotypeCaller/UnifiedGenotyper in GATK and VarScan2, applying a minimum of three variant-supporting reads and a mutant allele frequency (MAF) threshold of 0.5%. Common polymorphisms were filtered against dbSNP and the 1000 Genomes Project. Germline variants were excluded by comparison with matched whole-blood controls. An in-house database of recurrent sequencing artifacts, derived from >10,000 healthy individuals sequenced on the same platform, was used to further filter somatic calls. Gene fusions were identified using FACTERA, and copy number variations (CNVs) were analyzed with ADTEx. A log₂ ratio threshold of 2.0 was applied for copy number gains in tissue samples, while a threshold of 0.67 was used for losses in all samples; these thresholds were validated by droplet digital PCR (ddPCR)–based absolute copy number detection. Allele-specific CNV analysis was performed using FACETS, and segments with a drift threshold > 0.2 were classified as unstable. Chromosomal instability (CIN) proportion was calculated as the total length of drifted segments divided by the total genome segment length.

**ATAC-seq**

The service of ATAC-seq was provided by Active Motif Inc., using procedures as described previously [2]. In brief, 200,000 cells in duplicates were used for transposition reaction. After purified with MinElute PCR purification kit (Qiagen), transposed DNA was amplified by PCR with a total of twenty-five cycles, which was determined as optimal by qPCR. Libraries were then size selected using AMPure XP beads to remove fragments greater than 800 bp or smaller than 100 bp. The eluted library was validated for size distribution by DNA ScreenTape Assay (Agilent, USA) and quantified with Qubit dsDNA High-sensitivity Assay Kit (Thermo Fisher Scientific) on a Qubit Fluorometer. Finally, all the samples were pooled and sequenced on a NextSeq 500 in a pair-end modality rendering 2 x 150bp sequences. After removing low-quality reads and duplicate reads, the ATAC-seq data were aligned to the human reference genome hg38hg38 genome and the alignments were visualized on the UCSC genome browser.

**Antibodies**

The following antibodies were used for WB or IHC analysis: PARP1 (CST, 5625), caspase3 (CST, 9662), cleaved caspase3 (CST, 9664), Acetyl-Histone H3 (CST, 9649), Acetyl-Histone H4 (Abcam, ab177790) and SMARCB1 (santa cruz, sc-166165).

**Xenograft model**

For mice bearing Patient-derived Xenograft (PDX), primary Atypical Teratoid/Rhabdoid Tumor (AT/RT) samples from patient were collected with informed consent. The primary tumor fragments were implanted on the flanks of anaesthetized NSG mice, when the tumor reached 1-2cm3 (first generation), the tumor was segmented and reimplanted for passage. The fragments from third generation of PDX were implanted subcutaneously on the flank of female 6-week-old NSG mice. When the tumors reached a size greater than 100mm3, mice were randomly divided into four groups (n= 4): the control group received vehicle, the treatment groups received LBH589 or OTX015 or their combination at the same dose schedule as in mice bearing A204 xenograft. All animal experiments were approved by the institutional ethical review boards of the Renji Hospital affiliated Shanghai Jiao Tong University School of Medicine.

**References:**

1.	Shen Y, Zhao L, Li A, Peng Q, Liu Q, Wang L, et al. Rare myxoid pleomorphic liposarcoma: A case report and literature review. J Clin Pathol. 2024;77:358-62.

2.	Buenrostro JD, Giresi PG, Zaba LC, Chang HY, Greenleaf WJ. Transposition of native chromatin for fast and sensitive epigenomic profiling of open chromatin, DNA-binding proteins and nucleosome position. Nat Methods. 2013;10:1213-8.

**Supplementary Figure Legends**

**Figure S1. Transcriptional profiling of A204 cells treated with LBH589 and/or OTX015.**
**(A)** Principal component analysis (PCA) of RNA‑sequencing data from A204 cells treated for 16 h with DMSO (control), LBH589, OTX015, or the combination of both drugs. Each condition includes three biological replicates. **(B)** Volcano plots showing differentially expressed genes after treatment with LBH589 (left), OTX015 (middle), or the combination (right), compared to the DMSO control. Grey dots denote transcripts without significant change; red and blue dots indicate up‑regulated and down‑regulated transcripts, respectively. **(C)** Venn diagram illustrating the overlap of differentially expressed genes across the three treatment conditions. **(D, E)** Heatmaps displaying differentially expressed genes associated with the S‑phase **(D)** and G2/M‑phase **(E)** of the cell cycle.

**Figure S2. Transcriptional response of G401 cells to LBH589 or OTX015 treatment.**

**(A)** Principal component analysis (PCA) of RNA‑sequencing data from G401 cells treated for 16 h with DMSO (control), LBH589, or OTX015. Three biological replicates were included per condition. **(B)** Volcano plots showing differentially expressed genes after treatment with LBH589 (left) or OTX015 (right) relative to the DMSO control. Grey dots indicate transcripts without significant change; red and blue dots denote up‑regulated and down‑regulated transcripts, respectively. **(C)** Venn diagram comparing differentially expressed genes between LBH589 and OTX015 treatments. **(D, E)** Gene set enrichment pathway analysis of significantly down‑regulated genes after 16 h treatment with LBH589 **(D)** or OTX015 **(E)**. **(F, G)** Transcription‑factor motif enrichment analysis based on GSEA for LBH589 **(F)** or OTX015 **(G)** treatment compared to the DMSO control.

**Figure S3. Dual HDAC and BET inhibition cooperatively induces apoptosis in G401 cells.** A204 and G401 cells were treated with the indicated concentrations of LBH589 or OTX015, alone or in combination, for 24 h. Whole-cell lysates were then prepared and subjected to Western blot analysis to assess the protein expression levels of PARP1, caspase3 and GAPDH.

**Figure S4.** (**A**) Immunoblot analysis of Acetyl-Histone H3 and H4 in A204 cells treated with 35 nM LBH589 for indicated time period. (**B**) Time‑dependent downregulation of E2F1 and cyclin A2. A204 cells were treated with 35 nM LBH589 or 350 nM OTX015for the indicated time.

**Figure S5. Suppression of E2F1 activity by combined HDAC and BET inhibition.**
**(A)** Heatmaps displaying expression patterns of E2F1 target genes involved in cell cycle, apoptosis, DNA damage response, metabolism, and other cellular processes, based on RNA‑seq data from Figure 2. **(B, C)** RT‑qPCR quantification of E2F1‑regulated cell‑cycle genes in A204 **(B)** and G401 **(C)** cells after 16 h treatment with LBH589 and/or OTX015.

**Figure S6. Epigenetic modulation by LBH589 and OTX015 in MRT cells.** (**A, B**) Heatmap showing ATAC-seq coverage at sites with differential chromatin accessibility in LBH589- and/or OTX015-treated A204 (**A**) and G401 (**B**) cells compared to DMSO group. (**C**) Results of ATAC-seq in peaks at the genomic regions of *E2F1*, *CCNA2* and *CCNB1*. (**D, E**) Quantification of nascent *GAPDH, E2F1, CCNA2* and *CCNB1* mRNAs in LBH589- and/or OTX015-treated A204 (**D**) and G401 (**E**) cells. EU-labeled mRNAs were quantified by qRT-PCR. (**F**) Results of ATAC-seq in peaks at MYC locus, including the MYC promoter and three distal regulatory regions. (**G**) Immunoblot analysis of MYC in MRT cells treated with the indicated concentration of LBH589 and/or OTX015.

**Figure S7. *In vivo* suppression of E2F1 target genes by combined LBH589 and OTX015 treatment.** (**A**) Expression of the E2F1‑regulated genes CCNE2, CDK1, CDK2, CDC6, and E2F2 was measured by RT‑qPCR in A204 xenograft tumors from the indicated treatment groups. mRNA levels were normalized to GAPDH. (**B**) The expression of E2F1, cyclin A2, and cyclin B1 was quantified using an H-score (ranging from 0 to 300). This H - score was derived by multiplying a four-value staining intensity score (0, none; 1, weak; 2, moderate; 3, strong) by the percentage of cells showing positivity (0–100%). The bar graphs are presented as mean ± SD (n = 5). Statistical significance was determined by one‑way ANOVA with Tukey's multiple comparisons test; *P < 0.05,**P < 0.01, ***P < 0.001

**Figure S8. Expression levels of E2F1 in different types of sarcoma.** Representative H&E staining and E2F1 IHC in primary tumor samples from sarcoma patients. Scale bar, 50 µm.

**Figure S9. The combined inhibition of HDAC and BET exerts a synergistic effect on suppressing the in vivo growth of AT/RT PDX.** (**A**) Representative H&E staining and IHC for INI1 (SMARCB1) in AT/RT PDX and patient’s primary tumor sections. Scale bars, 50 µm. (**B**) Schematic of the in vivo treatment regimen. Tumor growth curves **(C)** and **body weight** changes (**D**) in each treatment group (n = 4). Data points represent mean tumor volume ± SD. **(E)** Representative images of excised tumors from each group at the study endpoint.
